# Between attraction and avoidance: from perfume application to fragrance-free policies

**DOI:** 10.1186/s12302-020-00377-8

**Published:** 2020-07-17

**Authors:** Ursula Klaschka

**Affiliations:** University of Applied Sciences, Prittwitzstraße 10, 89075 Ulm, Germany

**Keywords:** Consumer behavior, Consumer product, Fragrance, Fragrance-free policy, Hazard communication, Indoor air quality, Risk awareness, Safety behavior

## Abstract

**Background:**

According to a national representative survey, 19.9% of the German population describe various adverse effects on personal health upon exposure to fragranced consumer products. This study investigates whether these fragrance-sensitive persons have a higher risk awareness compared to the general public, whether they show a different safety behavior concerning fragrances and whether they reduce exposure and hence risk.

**Results:**

The presence of fragrances can have a major impact on the participation in public activities. Half of the fragrance-sensitive persons have ever been prevented from going to some place to avoid exposure to fragrances. More than half of them prefer fragrance-free alternatives (products, laundry, hotels, airplanes, health care facilities, or workplaces), while there are also fragrance-sensitive individuals, who indicate to prefer fragranced products and spaces. Half of fragrance-sensitive persons use perfumes to feel themselves more attractive. Furthermore, there is a large number of persons who prefer fragrance-free alternatives without being fragrance-sensitive. Around half of the general population indicate not to use a fragranced product if they know that it emits hazardous air pollutants. This shows that health effects associated with the presence of fragrances proved to be one out of several factors, but not the only one, which influences attitudes towards fragrances and their usage. The answers given reveal the multitude of aspects influencing risk awareness and safety behavior. According to the survey results, 7.4 workdays were lost due to illness from fragranced product exposure in the workplace per person on average, with estimated personal economic costs of 14.5 * 10^9^ Euro/year in Germany.

**Conclusions:**

The high prevalence of persons who correlate their health effects with exposure to fragrances shows that existing risk communication instruments are too weak, even for people who are aware of a risk, like fragrance-sensitive persons. The data substantiate how important it is to respect cognitive dissonance, confirmation bias and the inadequacy of the deficit model in risk management. The issue of adverse health effects associated with fragrances has reached a dimension, which requires immediate action: The results of this study are strong supporting arguments in favor of fragrance-free policies.

## Background

There is ample evidence that today’s hazard and risk information tools do not lead to a sufficient safety behavior concerning the use of hazardous fragrance compounds.

In Germany, 19.9% of the population report one or more types of adverse health effects from exposure to fragranced products [1]. In similar representative surveys in the USA, Australia, the UK, and Sweden the numbers were even higher with 34.7%, 33.0%, 27.8%, 33.1%, respectively, reporting fragrance sensitivity [2]. These results show the relevance of this issue.

The present study presents new data of the most recent nationally representative survey conducted in Germany [1, 3]. One main intention here was to find out whether there are differences between the fragrance-sensitive and non-sensitive persons in Germany. The hypothesis was that persons who link their health problems to the exposure to fragranced products have a higher risk awareness and hence show a more suitable safety behavior in comparison with the non-sensitive persons. Various existing hazard and risk communication strategies should fall on fertile ground and should motivate to reduce risk in such subpopulations. For example, one might expect that people who report health effects from fragrances would read the ingredient lists on the product containers, and this information would influence their purchasing and usage patterns. One might also expect that these persons would avoid fragranced spaces and prefer fragrance-free alternatives, such as fragrance-free hotels, airplanes or restrooms, subsequently.

It was shown previously, that there is a large support for fragrance-free spaces in countries analyzed [2, 4]. The present study examines whether fragrance-free alternatives receive support mainly from the fragrance-sensitive part of the population and from the persons who prefer fragrance-free products.

For these analyses, I used answers obtained by the representative survey conducted in Germany in 2019 [1, 3]. Surveys are an essential part to develop suitable hazard and risk communication strategies and they can complement the widely used “deficit model”. This model assumes that the transfer of information “top-down” from experts to the general public as recipients is sufficient to induce the appropriate safety behavior, an assumption which proved to be inadequate for a successful risk communication as shown by several studies [5–7]. For example, it was shown that the risk perception by experts and laymen differs largely [8]. In comparison, information obtained in surveys can be regarded as a “bottom-up” process with consultation and participation of the public. Such a “bottom-up” approach takes into account the target audience’s comprehension skills, their experiences, their personal preferences, cultural aspects and further parameters and helps to improve the effectiveness of “top-down” instruments [5]. Such a “two way” (“top-down” as well as “bottom-up”) communication process is urgent for complex issues, like the present one.

Based on these data, recommendations are deduced for an improved approach to fragrance use.

## Methods

The survey was effectuated as described in [1]. Using a random sample representative of age, gender, and region (n = 1102; confidence limit = 95%, margin of error = 3%), an on-line survey was conducted of the adult population (ages 18–65) in Germany. The survey was run in German. The process of survey translation and implementation was performed by Survey Sampling International (SSI), a global survey research company and online panel provider. For details on panel development, participant recruitment, survey design, and implementation, see Additional file 1: SSI Methodologies and Additional file 2: Survey Methods. The survey completion rate was 83%, and all responses were anonymous. Survey questions investigated the following areas: fragranced product use and exposure; health effects associated with exposure to fragranced products; specific exposure situations; effects of fragranced product exposure in the workplace and in society; preferences for fragrance-free environments and policies; and demographic information.

The following questions were added for the first time to the questionnaire that had been used in previous national surveys [9–12]: “Do you use perfumed products, such as perfume or deodorant, to make yourself feel more attractive?” “Do you consider people who use perfumed personal care products more attractive than people who do not?” “Do you consider people who use perfumed personal care products more hygiene-conscious than people who do not?” “Do you prefer that your clothes smell of fragrances after washing or that they do not smell after washing (no added perfume or no added odor)?” “Do you read the references to the products you use to get information about the fragrances it contains?” “Does the information about a particular fragrance ingredient in a product affect your purchasing decision?” “Do you believe that products with natural fragrance ingredients are healthier than products with synthetic fragrance ingredients?” “Do you prefer fragrance-free products when they are available?”

“Fragrance-sensitive persons” were defined here as persons who reported one or more types of adverse health effects from exposure to one or more types of fragranced products.

For the evaluation, I identified “subgroups” of people who answered “yes” to a specific question in the survey, e.g. all people who preferred fragrance-free products or all persons who used fragrances to feel more attractive.

Limitations of the study included the following: (a) it was not feasible to mention all possible product types and health effects. However, the low percentages for responses in the “other” category indicated that the survey captured the primary products and effects, although it cannot be excluded that the given list of choices creates a bias towards easy-to-select options. (b) Long-term health effects as well as health effects, which consumers could not link directly with the exposure to fragranced products, could not be considered here. (c) Data were based on self-reported data. This is the nature of the standard method of a survey. (d) The cross-sectional design of the study, which is useful for determining prevalence, is limited in the ability to determine temporal relationships and trends. (e) The survey focused on adults aged 18–65, which excluded data on effects of fragranced consumer products on children and the elderly, but allowed to obtain a picture of adult persons who may be in contact with fragranced products from their private use in addition to their workplaces. Children as especially vulnerable group of the population were not considered, because they would not be able to answer the questionnaire themselves. (f) Questions about exposure to various household products did not comprise information about the amounts applied, as it was not the purpose here to make a quantitative analysis of exposure. (g) It is the nature of a survey that it did not cover non-conscious health effects of scent. (h) The focus on fragrances and the detailed list of health effects in this survey might have led survey participants into temptation to relate their health effects to fragrances, where there might not have been any link. (i) Furthermore, it depends on the application pattern of a fragranced product how severe consumers perceive risk (e.g., perfume versus lotion). (j) Prolonged high concentrations of perfumes in the breathing air reduce the ability to sense the odors due to olfactory adaptation. Under these conditions, persons might experience health effects due to fragrances, but cannot relate them to fragrance exposure because they do not perceive the odor. (k) As the perception of fragrances depends on the awareness of fragrances and the sense of smell, fragrance-sensitive persons will be aware of fragrances in a room at very low concentrations, whereas a scented room may be perceived as “fragrance-free” by people who are adapted to elevated fragrance concentrations. This potential bias of the comprehension of “fragrance-free” spaces cannot be avoided in such a survey.

## Results and discussion

### Many consumers use perfume to feel attractive

The focus here is on fragrance-sensitive persons and on persons who prefer fragrance-free products. These subgroups are not congruent, but they overlap: two-thirds (62.6%) of fragrance-sensitive persons prefer fragrance-free products, and a third (29.9%) of the subgroup of persons who prefer fragrance-free products are fragrance-sensitive. Nearly three-fourths (72.5%) of the general population and more than half (54.8%) of fragrance-sensitive people state that they use perfumed personal care products to feel themselves more attractive. Nearly two-thirds (62.2%) of people who prefer fragrance-free products use them to feel themselves more attractive (Table 1).

**Table 1:** Fragrances and personal attractiveness

	General population	People who prefer fragrance-free products	People who do not prefer fragrance-free products	Fragrance-sensitive people	Non-fragrance-sensitive people
Number of persons in (sub)groups	1102	458	644	219	883
Do you use perfumed products, such as perfume or deodorant, to make you feel more attractive?	72.5	62.2	79.8	54.8	76.9
Do you consider people who use perfumed personal care products to be more attractive than people who do not?	45.4	40.0	49.2	35.6	47.8
Do you consider people who use perfumed personal care products to be more hygiene-conscious than people who do not?	37.8	33.8	40.7	31.1	39.5

Percentage of “Yes” answers given to the questions in column 1 by the general population and the various subgroups. (Sizes of groups see first line.)

In all groups, the percentage of people who state that the application of perfumed personal care products increases their own attractiveness is higher, compared to the percentages of the participants who state that these products increase the attractiveness of someone else. Nearly half of the general population (44.8%) consider being next to someone wearing a perfumed product, such as perfume or aftershave lotion, generally more attractive, while 39.1% are not sure, and 15.2% think of them to be more repelling. More than a third (36%) of persons who prefer fragrance-free products consider being next to someone wearing a perfumed product more attractive, while 41.5% are not sure and 21.6% consider it more repelling. Less than a third (28.8%) of fragrance-sensitive persons consider being next to someone wearing a perfumed product more attractive, while 34.7% are not sure and also 34.7% consider it more repelling.

Up two-fifths of all groups (31.1–40.7%) consider people who use perfumed products to be more hygiene-conscious than people who do not (Table 1).

These findings have the following implications:

One of my hypotheses was that people who prefer fragrance-free products would not use perfumed products themselves to feel more attractive. However, nearly two-thirds of them do so. Two-fifths of this subgroup even consider people who use perfumed products more attractive, about the same number of this subgroup is not sure, and more than a third of this subgroup consider being next to someone wearing a perfumed product more attractive. At first sight, this seems to be counterintuitive and an example of cognitive dissonance. This shows that the situation is not as easy as expected. Further information is needed to understand why people give these answers. For example, it is possible that the participants have different perceptions of the term “attractive”. The answers could also be due to an indirect effect, because people wearing perfume are likely to take care of their appearance, like good and attractive clothing, makeup, hairstyle, and send other more subtle signals by their behavior. Furthermore, one could imagine that people who prefer fragrance-free products in general would nevertheless accept fragrances in some special product types, for example in personal care products and perfume. In addition, the different attitudes towards own perfume use and perfume applied by others might be due to different subjective personal preferences for various scents and concentrations.

Another hypothesis of mine was that people in the subgroup, who answered that they use perfumed personal care products to make themselves feel more attractive, would consider the proximity to a fragranced person also more attractive, but this hypothesis is wrong. Only half of these people (56.3%) are of this opinion and 8.9% consider this even more repelling. This might again be explained by different subjective personal preferences for various scents and concentrations. Furthermore, people who use the same perfume over many years tend to increase the amounts applied due to olfactory adaptation, whereas other perfumes are easily sensed and might cause repellence.

In addition, I presumed that less fragrance-sensitive persons would use perfumed products to feel themselves more attractive, because I assumed that fragrance-sensitive persons associate their health effects with fragrances and they would at least reduce the exposure from their own use. There is a clear difference between the sensitive and the non-sensitive group, but I had expected more. Half of the people, who correlate their health effects with fragrances, use fragrances to feel themselves more attractive. This means that there must be important reasons why many people accept negative health experiences for the sake of feeling attractive. In the case of perfumes, the promise to become attractive is very prominent in the advertisements. Toncar and Fetscherin analyzed the visual puffery in the ubiquitous use of imagery-laden ads in the promotion of personal fragrances [13]. They describe that fragrances might be “a fantasy product, intimately connected to the self-esteem or self-image and perceived desirability and attractiveness of the buyer. Consumers are not buying the fragrance alone, but the imagery that becomes intimately associated with the fragrance.” [13]. The data in our study suggest that such promises outweigh the health effects experienced by fragrance exposure.

My next hypothesis was based on the growing practice of applying fragrances with “air fresheners” in elder care facilities to mask unpleasant odors [14]. I presumed that some people confound “fragranced” with “clean” or “hygienic” and vice versa and hence correlate the smell of fragrances with cleaning, because the presence of fragrances without previous cleaning could result in a similar odor. Indirect effects might again play a role, as the notion of “hygienic” is based on personal experience where the visual cleanliness and orderliness of a space often goes along with a pleasant smell. In the present study, more than a third of the general population correlate the smell of perfume with hygiene. This false sense of hygiene may wrongly encourage individuals to become negligent with their safety behavior. The strategy to utilize fragrances instead of applying hygienic measures increases the indoor concentration of hazardous substances and, in addition, insufficient hygienic standards increase biological risks.

### Repellent places: fragranced indoor air makes people leave

Does fragranced indoor air influence fragrance-sensitive people’s participation in society?

Table 2 shows examples where people are reluctant to stay in places away from their homes because of scented air. More than half of the fragrance-sensitive persons (51.1%) have ever been prevented from going to some place because they would be exposed to a fragrance product that would make them sick. One out of ten (9.7%) non-fragrance-sensitive persons indicate this, too. Nearly half of the fragrance-sensitive persons (48.4%) indicate that if they enter a business and smell air fresheners or some fragranced product, they would want to leave as quickly as possible, compared to 5.7% of non-fragrance-sensitive persons. Two-fifths (43.8%) of fragrance-sensitive persons indicate to have been unable or reluctant to use the toilets in a public place, because of the presence of a scented product, compared to 9.1% of non-fragrance-sensitive persons. More than a quarter (27.4%) of the fragrance-sensitive persons have ever been unable or reluctant to wash their hands with soap in a public place, because they knew or suspected that the soap was fragranced, compared to 3.6% of non-fragrance-sensitive persons.

**Table 2:** Repellent places

	General population	Fragrance-sensitive people	Non-fragrance-sensitive people
Have you ever been prevented from going to some place because you would be exposed to a fragrance product that would make you sick?	18.0	51.1	9.7
If you enter a business, and you smell air fresheners or some fragranced product, do you want to leave as quickly as possible?	14.2	48.4	5.7
Have you ever been unable or reluctant to use the toilets in a public place, because of the presence of an air freshener, deodorizer, or scented product?	16.0	43.8	9.1
Have you ever been unable or reluctant to wash your hands with soap in a public place, because you know or suspect that the soap is fragranced?	8.3	27.4	3.6

Percentage of “Yes” answers given by the general population and by (non-)-fragrance-sensitive persons to the questions in column 1. (Sizes of groups see first line in Table 1.)

These results have the following implications:

My hypothesis was that people who report health effects from fragrances would avoid fragranced spaces. A large fraction of fragrance-sensitive persons indicate to avoid the fragranced spaces in question, but the results are not as clear as expected, as also many persons who do not indicate to have health effects from fragrances give these answers, too. These results can be influenced by the different awareness of fragrances: fragrance-sensitive persons will be aware of fragrances in a room at very low concentrations, whereas a scented room may be perceived as “fragrance-free” by people who are adapted to elevated fragrance concentrations. The data do not allow to decide whether the non-avoidance was a conscious decision or a lack of perception.

The examples show that there are heavy curtailments for the daily lives of persons who are reluctant to use public toilets or other places due to fragranced air. The presence of fragrances in the breathing air makes their participation in cultural or social activities difficult. It is very important to note, that even a considerable number of non-fragrance-sensitive persons indicate to avoid fragrances in the public places in question and live with such constraints. This shows that the health problems associated with fragrances are not the only motivating cause for this avoidance behavior.

### Knowledge on fragrances has some impact on user behavior

Nearly a third of the general population (28.3%) indicate to read the references to the products to get information about the fragrances it contains. In comparison, almost half of the people who are fragrance-sensitive (44.7%) indicate to read the references to the products.

A third (31.8%) of the general population say that the information about a particular fragrance ingredient in a product would affect their purchasing decision. In comparison, half of the fragrance-sensitive (54.3%) group and two-thirds (66.3%) of the subgroup of people, who indicate to read the references, state that the information about a particular fragrance ingredient in a product would affect their purchasing decision.

Nearly half (48.2%) of the general population state that they would not use a fragranced product, if they knew that the product emitted hazardous air pollutants. In this case, the difference between fragrance-sensitive persons (58.4%) and non-fragrance-sensitive persons (45.6%) who gave this answer is not very pronounced.

The data show, that basic knowledge about the chemistry of fragrances has some impact on user behavior.

My hypothesis was that fragrance-sensitive people would tend to read the references on the products to get information about fragrance ingredients, so that they could adapt their behavior correspondingly. However, less than half of them do so. One potential explanation could be that many fragrance-sensitive persons do not read labels because they have developed a high risk awareness and know that labels are generally incomplete and often inaccurate [15], and therefore might provide misleading and unhelpful information. Another potential explanation could be that these persons have already made the efforts of a detailed selection of a set of products, which correspond to their needs and which they keep buying. Thus they do not need to check out any other product and do not need to read the ingredient lists on the labels of any other products. A quarter of the people who are not fragrance-sensitive indicate to read the references on the products to get information about fragrance ingredients. This compares with my results obtained in a survey with interested and motivated consumers conducted in 2016 where more than three quarters indicated to use hazard pictograms, information on the packaging, and list of ingredients as preferred information sources on products [16].

My next hypothesis was that fragrance-sensitive people would refrain from buying a product if they received the information about a particular fragrance ingredient. A representative survey conducted in Germany in 2010 asked about factors influencing the purchase of a product. The survey participants indicated, when they were asked about personal care products, that information on the ingredients had more influence on their choice of a product than selling price and personal experience compared to other consumer goods, where consumption choices are mostly price-driven [17]. A multitude of further aspects influence customers in making their purchasing choices, such as quality, environmental aspects, efficiency, test results published by consumer organizations, product brand, odor, and safety information [17]. The results in my survey show, that half of the fragrance-sensitive persons and a third of the general population use the information about a fragrance ingredient for their purchasing decisions. This means that the information about a fragrance ingredient can be regarded as one single very relevant factor in the process of consumers’ product selection compared to the other influencing aspects.

Furthermore, I had the hypothesis, that more fragrance-sensitive persons would clearly refrain from using fragranced products that emitted hazardous air pollutants, but the numbers are only slightly higher than the average. These results should be seen in the light of the answers given by the survey participants on their exposure to fragrances. Nearly all of the general population (96.3–99.1%) indicate to expose themselves to fragrances [3], while at the same time around half of the population indicate that they do not use a fragranced product, if they learned that it emitted hazardous air pollutants. This fact might be explained by the insufficient knowledge about the general presence of hazardous air pollutants in fragranced products. It is also an example for cognitive dissonance and for the inadequacy of the deficit model: Most consumers do not change their fragrance use, even if they know about the hazardous properties, because many other aspects influence their behavior.

### Many consumers prefer fragrance-free alternatives

Previous national surveys revealed that fragrance-free environments receive a major support [2, 4].

A third (36.5%) of the general population prefers that their clothes do not smell of fragrances after washing, while every second person (48.1%) prefers that they smell of perfume after washing. More than half (52.4%) of the individuals who prefer fragrance-free products do not want that their clothes smell of fragrances after washing, while more than a third (35.2%) prefer the smell of perfume. The numbers are in the same range for fragrance-sensitive persons (50.2% prefer no perfume, 38.4% prefer the smell of perfume in their laundry) (Table 3).

**Table 3:** Preference of fragrance-free products

	General population	People who prefer fragrance-free products	People who do not prefer fragrance-free products	Fragrance-sensitive people	Non-fragrance-sensitive people
Preference of clothes with or without smell after washing					
No–no smell after washing	36.5	52.4	25.2	50.2	33.1
Not sure	15.0	12.2	16.9	11.0	16.0
Yes—smells of perfume after washing	48.1	35.2	57.3	38.4	50.5
Preference of fragrance-free products					
Yes	41.6	100	0	62.6	36.4
Not sure	24.8	0	42.4	14.6	27.3
No	33.4	0	57.1	22.4	36.1

Percentage of answers given by the general population and the various subgroups to the question “Do you prefer that your clothes smell of fragrances after washing or that they do not smell after washing (no added perfume or no added odor)?” “Do you prefer fragrance-free products when they are available?” (Sizes of subgroups see first line in Table 1.)

Two-fifths (41.6%) of the general population prefer fragrance-free products when they are available (Table 3). The demographic group with the highest percentage of people who prefer fragrance-free products are men aged 55–65 (54.9%). The demographic group with the lowest percentage of people who prefer fragrance-free products are women of the same age group 55–65 (29.2%). In all other age groups, the differences between the preferences of the genders are not significant.

More than half of the general population (58.4%) prefer hotels without fragranced air. The percentages are higher for persons who prefer fragrance-free products (70.7%) and for fragrance-sensitive persons (69.9%) (Table 4). (Note that these two groups overlap to some extent: two-thirds of fragrance-sensitive persons prefer fragrance-free products.)

**Table 4:** Preference for fragrance-free environments

	General population	People who prefer fragrance-free products	People who do not prefer fragrance-free products	Fragrance-sensitive people	Non-fragrance-sensitive people
Hotel without fragranced air					
Yes (without fragranced air)	58.4	70.7	49.7	69.9	55.6
Not sure	23.1	13.5	30.0	12.3	25.8
No (with fragranced air)	18.1	15.5	20.0	16.4	18.6
Airplane without fragranced air					
Yes (without scented air)	57.0	67.7	49.4	70.3	53.7
Not sure	26.7	18.8	32.3	14.6	29.7
No (with scented air)	15.7	13.1	17.5	13.2	16.3

Percentage of answers given by the general population and the various subgroups to the questions: “Staying in a hotel with/without fragranced air. Which would you choose?” and “Flying on an airplane that pumped/did not pump scented air throughout the passenger cabin. Which would you choose?” (Sizes of (sub)groups see first line in Table 1.)

More than half of the general population (57.0%) prefer flying on an airplane without scented air. The percentages are again higher for persons who prefer fragrance-free products (67.7%) and for fragrance-sensitive persons (70.3%) (Table 4).

The preferences for non-scented hotels (average 60.7%) and for non-scented airplanes (average 62.5%) were slightly higher in the multinational study conducted in the USA, Australia, UK, and Sweden [2] compared to the data in obtained in Germany (non-scented hotels (58.4%), non-scented airplanes (57.0%)).

These findings have the following implications.

My hypothesis was that all persons in the subgroup, who declare to prefer fragrance-free products, would want their laundry without fragrance and choose hotels and airplanes without scent, but this is not the case. There is a large number in the complementary subgroup of people who do not declare to prefer fragrance-free products, but who prefer fragrance-free hotels and airplanes. At the same time, there are some people who declare to prefer fragrance-free products, but nevertheless they choose the option of hotels with fragranced air (15.5%) and prefer flying on an airplane that pumps scented air throughout the passenger cabin (13.1%). One could assume that persons who read the labels want to have the control about fragrances in their private use and would have higher distrust to the exposure by others in fragranced spaces, but this was not as evident as expected. Only slightly more persons in the subgroup who read the labels (63.1%, and 61.9%) compared to the average prefer hotels or airplanes without fragrances (data not included in Table 4).

I had also expected that predominantly fragrance-sensitive persons would choose fragrance-free alternatives, but this is also not the case, as there is a large number of persons, who prefer fragrance-free alternatives without being fragrance-sensitive. And at the same time, more than a third of fragrance-sensitive persons choose perfume in their laundry, one out of six choose a hotel with fragranced air and one out of eight choose airplanes that pump scented air through the passenger cabin. I assume that especially people who have not experienced hotels or airplanes with fragranced air themselves might consider a scented environment as quality feature and answer therefore that they prefer the scented alternatives or are not sure what to answer.

The large numbers of persons who are not sure what to answer (up to 32.3% in the subgroups considered) can be seen as an indication that it was not easy for the participants to decide, because there are additional factors, that influence their decisions. Some survey participants might have had difficulties answering these questions because they did not know what information could help them to avoid fragranced airplanes or hotels before they enter the plane or the hotel. There is no general certification or information tool about the presence of fragrances in indoor air in Germany. Even “green key” certified hotels sometimes use fragrances. Another reason for the difficulty to decide could be that the individuals tried already to find fragrance-free alternatives but could not find any. This was for example the case for patients with fragrance contact allergy in a previous study [18]. A similar ambiguity between desired effect and potential toxic side effects that influences the attitude of consumers was also described for the general use of household chemicals [17]. This compares also to the complex and equivocal situations known from studies on manipulative odor marketing [19, 20]. These data reveal again that survey participants give answers, which seem at first sight counterintuitive, but might be perfectly rational for reasons, which were not addressed in the survey. Some potential further explanations are also discussed in [3].

### Fragrance-free policies are preferred

Who is in favor of fragrance-free policies in the German population?

Nearly half of the general public (46.4%) prefer that health care facilities and health care professionals be fragrance-free. Nearly two-thirds (63.9%) of the fragrance-sensitive group have this preference. The highest support for any of the three fragrance-free options in Table 5 comes from people who prefer fragrance-free products, where two-thirds (66.2%) are for fragrance-free health care facilities (Table 5).

**Table 5:** Preference of fragrance-free policies

	General population	People who prefer fragrance-free products	People who do not prefer fragrance-free products	Fragrance-sensitive people	Non-fragrance-sensitive people
Fragrance-free health care facilities					
Yes	46.4	66.2	32.3	63.9	42.0
Not sure	23.4	14.2	30.0	11.9	26.3
No	29.8	19.4	37.1	23.3	31.4
Fragrance-free workplaces					
Yes	33.2	51.5	20.2	54.3	28.0
Not sure	35.3	26.0	41.9	22.4	38.5
No	30.6	22.3	36.5	22.4	32.6
Fragrance-free restrooms					
Yes	21.6	36.7	10.9	45.7	15.6
Not sure	28.2	25.5	30.1	26.0	28.8
No	50.0	37.6	58.9	28.3	55.4

Percentage of answers given by the general population and the various subgroups to the questions: “Would you prefer that health care facilities and health care professionals be fragrance-free?” “Would you be supportive of a fragrance-free policy in the workplace? And “Would you be supportive of a ban on air fresheners in restrooms and toilets?” (Sizes of subgroups see first line in Table 1.)

A third (33.2%) of the general population is supportive of a fragrance-free policy in the workplace, while even more (35.2%) are not sure. More than half of fragrance-sensitive persons (54.3%), and of the people who prefer fragrance-free products (51.5%) are in favor of a fragrance-free working places (Table 5).

A fifth of the general population (21.6%) is in favor of a ban of air fresheners in restrooms. Nearly half of the fragrance-sensitive persons (45.7%) are in favor of fragrance-free restrooms. More than a third (36.7%) of people who prefer fragrance-free products are supportive of a ban on air fresheners in restrooms and toilets (Table 5).

Comparing the three fragrance-free scenarios in question, more persons are in favor of fragrance-free health care facilities compared to fragrance-free workplaces or restrooms in each subgroup (Table 5).

These findings have the following implications:

My hypothesis was that all persons in the subgroup, who declare to prefer fragrance-free products, would prefer fragrance-free policies, but this is not the case. There is a considerable number in the complementary subgroup of people who do not declare to prefer fragrance-free products, but who prefer fragrance-free policies. One reason for this could be that people have a feeling of control with their own products while they are uncomfortable about not being able to control the exposure by others, e.g., in perfumed spaces. Another explanation might be based on a financial aspect: as the amounts needed for continuously scenting air in facilities are large, the manager might use products of minor quality which do not match a broad audience and hence such fragrances might be perceived as rather disturbing.

The preferences for fragrance-free workplaces and for fragrance-free health care facilities are somewhat smaller in Germany compared to the data obtained in the above-mentioned international studies [average preferences for fragrance-free workplaces (47.8%) and fragrance-free health care facilities (51.4%)] [2]. In addition, the percentage of fragrance-sensitive persons is also lower in Germany (19.9%) [1] compared to other countries (average 32.2%) [2]. These data were the reason for me to ask whether especially fragrance-sensitive persons prefer fragrance-free options. This would explain why both the preferences for fragrance-free policies and the number of fragrance-sensitive persons were higher in other countries. Many fragrance-sensitive persons support any of the three fragrance-free options here, but there are also many non-fragrance-sensitive persons, who prefer these alternatives. This means that the lower number of supporters of fragrance-free alternatives is not necessarily due to a lower number of fragrance-sensitive persons.

The questions in Table 5 concerned situations that pertain also to fellow citizen (health care facilities, workplaces or restrooms), unlike the previous questions in Tables 3 and 4, which concern only the personal individual surroundings of the respective respondent. Therefore, the answers might be different, if people might not want to impose their personal preferences of a fragrance-free space on other people.

The number of persons, who are not sure, what to answer to these three questions is rather high (up to 41.9% in the subgroups) (Table 5) which may be seen as an indication about the various additional factors people would like to consider before making a definite decision. It is possible that they have not made experiences of their own with fragranced spaces so far. A survey with open questions where survey participants would be able to describe their preferences more precisely would yield more detailed information about their beliefs and attitudes.

The data confirm that there is strong demand for fragrance-free policies. In the United States and in Canada, there are already numerous fragrance-free buildings and organizations for example libraries, schools, universities, town facilities, or health care providers [4]. In Germany, there are many fragrance-free products on the market, especially personal care products recommended by the German Allergy and Asthma Association DAAB [21], whereas the author does not know of any explicit public fragrance-free spaces or buildings in Germany so far. Public restrooms are frequently scented in Germany. There are no public representative data about the prevalence of scented spaces in Germany, but some retailers that sell equipment for indoor odor marketing list their customers on their homepages [22, 23]. These lists show that odor marketing plays a certain role. Currently, the German Environment Agency supports a research project about fragranced in-door air in Germany [24].

### Economic costs due to fragrance exposure in the workplace are estimated to 14.8 Billion Euros per year in Germany

In the present survey nearly every forth fragrance-sensitive person (22.4%) reports to have become sick, have lost workdays or a job, in the past year, due to illness from fragranced product exposure in the workplace, compared to 9.4% of the subgroup who prefers fragrance-free products, and compared to 5.5% of the general population. According to the answers given, 7.4 workdays (8 h equivalent) were lost due to illness from fragranced product exposure in Germany per person on average. This number is higher than the average of 5.1 lost workdays per person in USA, Australia, Sweden and the United Kingdom together, although the percentage of self-reported fragrance-sensitive persons is lower in Germany (19.9%) compared to the average in the other countries (32.2%) [2]. The answers given by the survey participants allowed to estimate the costs resulting from lost income, medical expenses, and other costs over the last 12 months due to illnesses from fragranced products: 14.5 * 10^9^ Euro in Germany per year, which amounts to 0.4% of the annual German gross domestic product in 2019 [25]. This calculation is based on the self-reported numbers in the representative survey in 2019 (see Additional file 3) and shows the vast economic impact. Costs for the employers and the public health system caused by fragrance-induced illnesses are not known (Table 6).

**Table 6:** Economic aspects

	Million Euro
Estimated personal economic costs due to fragranced product exposure in workplace amounts in Germany in 1 year	14500
Annual sales for women’s fragrance products in Germany in 2018 [28]	976
annual sales for men’s fragrance products in Germany in 2018 [28]	516
Annual sales for deodorants in Germany in 2018 [28]	757
Annual expenses for commercials on perfumes and fragranced products in Germany 2017 [29]	278

Economic impact of costs resulting from lost workdays and lost jobs due to illnesses from fragranced products amounts in relation to some selected numbers concerning economic aspects

As comparison, the estimated annual costs caused by tobacco consumption amount to 97.24 * 10^9^ Euro in Germany, with a third being direct health related costs, and two-thirds indirect costs for the economy due to production loss [26]. Lost workdays due to tobacco consumption amount to around 35.9 * 10^9^ Euro in Germany per year (permanent and temporary disability) [26]. As these very detailed data were raised by different methods [27], they cannot be compared directly with the numbers calculated from the survey data.

It is worthwhile to place these numbers next to the increasing sales numbers for perfumed products. For example, the annual sales in Germany amounted to 976 million Euro for women’s fragrance products, to 516 million Euro for men’s fragrance products and to 757 million Euro for deodorants in 2018. Other fragranced products such as air fresheners or cleaning supplies are not included in these numbers [28].

In comparison, the official statistical data concerning annual expenses for commercials for perfumes and fragranced products reached more than 278 million Euros in Germany in the year 2017 [29] (compared to 206 million Euros for tobacco advertisement in Germany in 2013 [30]). The expenses for the advertisement for perfumes is around fourfold compared to the production costs [31].

I do not have the means to quantify other economic aspects of fragrances, such as for example job market impact on the domestic economy. Nevertheless, the numbers described give some indication about the economic impacts of fragrances for the German society, as well as for the producers, retailers, advertisers, health care professionals, as for fragrance-sensitive persons, their families and employers.

### The long way from hazard communication to safety behavior

The key message of the data presented confirm previous findings that show that today’s hazard and risk information tools for fragrances do not lead to a sufficient safety behavior.

Some results in the present analysis concern the usage of *hazard communication instruments*: e.g., reading the references to know more about a specific fragrance ingredient. Other results deal with *hazard and risk awareness*: e.g., the health effects reported by fragrance-sensitive persons, and further results are indications for *safety behavior*: e.g., fragrance application, avoidance reactions of fragranced spaces or preference for fragrance-free products.

There are a multitude of *hazard and risk information instruments* designed to increase knowledge in consumers [32]. (Note: Hazard communication is closely linked to risk communication. A hazard is defined as ‘a possible source of danger’, described through standardized classification and labeling [33], while the level of risk is dependent on hazard in combination with exposure [34].) Existing hazard communication instruments, such as compulsory ingredient lists on personal care, washing and cleaning products, information campaigns by federal authorities (such as the German Federal Environment Agency), information by consumer organizations (such as the German Allergy and Asthma Association, DAAB), scientific publications, or internet forums provide information needed for motivating consumers to a suitable safety behavior. Another example are hazard pictograms which are widely known and trusted [16]. My results obtained in a survey with interested and motivated consumers in 2016 showed that more than three quarters declared to use hazard pictograms, information on the packaging, and list of ingredients as preferred information sources on products [16]. Nearly all (91%) consumers in Germany indicated to recognize chemical risks through the hazard labels on the product in general [17]. In a survey conducted by the European Union, 72% of the German population said that they would use warning symbols to find out whether a product is hazardous [35]. However, the high incidence of adverse health effects induced by fragrance ingredients illustrates that current legal measures and *hazard and risk communication* efforts fail to protect consumers from hazardous exposures sufficiently. *Hazard and risk communication* instruments are not always as effective as expected, as the following examples illustrate. Most consumers are not informed about sources of fragrance exposure and the (eco)toxicology of fragrances [3, 34]. Furthermore, the precise comprehension of hazard labels is rather poor in the general population in Europe [35], even if the majority indicates to use this hazard communication instrument as described above. Many ingredients in personal care products (fragrance substances as well as other compounds) are classified as hazardous substances [36], but personal care products are exempt from hazard labeling. This means that the products do not need to be labeled as hazardous mixtures even if their hazardous ingredients are present in amounts above the thresholds for labeling [37]. Most consumers do not know this. Furthermore, it was shown, that the mandatory list of 26 fragrance ingredients on personal care products cannot be regarded as an effective hazard communication method, as consumers are unable to understand the implications [34, 36] and many hazardous fragrance ingredients are not listed. Ingredient lists may be regarded as hazard communication tool, but several studies show that by law not all relevant ingredients need to be declared [34] and that some ingredients are not listed although they are present in relevant concentrations [15]. In addition, wrong assumptions about fragrance compounds are frequent [3]. In general, the public has limited knowledge about ingredients in consumer products and the potential health effects of these substances [18, 35, 38]. The data in the present study show that fragrance-sensitive persons do not rely on ingredient lists, as only half of them indicate to read them. This supports the finding that ingredient lists are not an efficient hazard communication tool, even for affected persons.

There are a multitude of factors which influence consumers’ *safety behavior*, some of which are relevant for the use of fragrances and are mentioned here. A representative survey in Germany [17] found that consumers considered products which they use frequently (such as personal care products or other fragranced household products) as less dangerous compared to products which they used only once in a while. Beautiful messages and promises in the commercials for fragranced products might mislead consumers [13] and counteract warnings. Many of today’s advertisements use easily comprehensible messages that refer to current moral or quality expectations of consumers, such as “organic”, “fair trade”, “no palm oil”, “no nanotech”, “no parabens”, “no aluminium” and so on. Furthermore, scents are special: People are readily prone to interpret certain odors. In a survey conducted by the European Union, 22% of the German population said that they would recognize from the smell of a product whether a product is hazardous [35]. In a survey among motivated and interested German persons, also 15.8% said that they would use the smell of a product to find out whether it contains hazardous substances. These answers were independent of the person’s knowledge in chemistry [16]. The question is, how do people recognize chemical hazard by the smell? Fragrance ingredients can induce positive associations, while most of the frequently used compounds are classified as hazardous substances [36]. It must be questioned whether people who use their olfactory sense to discern hazardous products, are aware of this fact. This example shows very nicely the three essential aspects, which make risk communication so difficult: cognitive dissonance (adhering to contradicting beliefs without realizing it), confirmation bias (searching for information that confirm own beliefs) and the trust in the deficit model (believing that increased knowledge would improve safety behavior automatically). Another aspect is, that many consumers trust in the safety of fragranced products: For example, it was shown that personal care products enjoy the highest trust compared to other household products [17]. These aspects of subjective interpretations of fragrance use show the complexity of hazard and risk communication. All efforts to educate the general public about the hazardous side of chemistry in every-day products need awareness of these psychological findings as decisive drivers for consumers’ safety behavior.

Table 7 visualizes the steps from *hazard and risk communication* to *safety behavior* with additional influencing factors concerning fragrances. This list is not complete, but shows some broader implications of the results in the present study.

**Table 7:** From hazard communication to safety behavior

**Hazard and risk communication instruments**	Information tools that increase knowledge (e.g., by authorities, consumer organizations, scientists, interested public) [16, 32, 35] Instructions for safe use [35] Information on the product (such as hazard pictograms or ingredient lists)* [16, 34, 35] Information in the public media [16, 32]
**Factors affecting risk awareness**	Observed health effects* Health effects observed in the family or with friends
**Factors affecting safety behavior**	**Physiological and psychological aspects:** Quality of perception of odors* Perception of a certain odor intensity Personal preferences and experiences* [13, 35] Positive or negative associations with certain odors* [13, 35] Subconscious effects [19, 20] Addiction to fragrances **Personal history:** Observed adverse health effects* Personal experiences [17, 35] Willingness to participate in normal societal life* Commonly applied fragrances in society [18] **Influence by others:** Attitude of friends, family, colleagues [16, 35] Advertising (promise to become attractive)* [13, 39] Product brands [17] **Assumptions and beliefs:** “Frequently used products are less dangerous” [17] “Natural substances are healthier than synthetic ones.”* [3, 16] “Pleasant smells are good for me.” [13] “Pleasant smell is an indication for hygiene.”* Trust in safety of products [16, 17, 40]

Examples for basic hazard and risk communication instruments and potential factors influencing risk awareness and safety behavior in the case of fragrances. Results in the present study concern the aspects labeled with an asterisk

The present data show that today’s *hazard and risk information* tools do not lead to a sufficient *safety behavior*. In principle, consumers should use all these factors for the interpretation of the risks by conducting a kind of risk assessment for their personal context when exposing themselves to these chemicals [34]. However, it is evident, that they are not able to do this properly. Instead, individuals might adopt a pragmatic attitude towards fragranced products, where vulnerable people might tolerate negative health effects for the sake of the other motives. This is again a good example for cognitive dissonance. An efficient *hazard communication* should consider these multiple potentially conflicting influences on consumers’ attitudes and their behavior. *Hazard communication* should not only transfer knowledge, but it should also aim at building trust. As explained above psychological aspects may be more important for risk communication than the pure transfer of knowledge. Public discussion could raise awareness and encourage the target groups to learn and participate in the risk management processes [40]. According to ethical standards for scientists, it is their responsibility to communicate their results whenever they are of relevance to the society like in the present case. This includes also the difficult task of improving communication about inherent uncertainties of scientific findings. The final goal of hazard and risk communication should always be to motivate for a suitable *safety behavior*.

Reducing exposures to fragrances would be a straightforward way to reduce observed health effects in consumers, which could provide benefits for the affected persons as well as for society. Non-chemical alternatives are most recommendable (e.g., better hygienic measures in health care facilities and toilets, fresh air from the outside by opening the windows instead of “air fresheners”). Fragrance-free spaces, especially in health care facilities and other public places could help to avoid and reduce exposure, which would help affected people to take part in public activities again.

As long as fragrance-free alternatives are scarce [4], the huge number of persons who describe adverse health effects upon exposure to fragranced products should be taken serious. Improved *hazard and risk communication* tools could reduce the number of affected persons. One step in this direction would be hazard symbols on the packaging. As long as personal care products are exempt from classification and labeling [33], consumers are misled by the lacking hazard labels on cosmetic containers [37] and even more by the seducing promises in the commercials and on the packaging [13]. Clear mandatory labels on all kinds of fragranced products, not only personal care products, would raise consumers’ awareness [41]. However, this step would certainly not be sufficient. The example of tobacco consumption shows, that cigarette sales did not drop much after introduction of the mandatory warning labels on packages in Germany, while the increase of the price per package over the years by means of raised taxes was much more effective [30]. In the case of fragrances, I assume that an increase in price would not be an effective measure. The data imply that a change in the public attitude towards fragrance use could make a difference. It is the responsibility of all parties involved to take action for the sake of consumers, and especially for the sake of the most vulnerable groups of the society such as children, elderly or sick people. It is worthwhile to improve the various steps in the long way from *hazard communication* to *safety behavior*.

## Conclusions

There are strong supporting arguments in favor of fragrance-free policies:

- A large part of the population report adverse health effects from exposure to fragranced products.
- The impact of fragrances on the environmental organisms is relevant [42].
- A large number of persons are prevented from going to public places because they would be exposed to fragranced products that would make them sick and are therefore excluded from participating in social life.
- People who observe adverse health effects upon exposure to fragrance presumably are aware of a risk, but apparently, there are many additional aspects, which influence their attitudes and behavior. There seems to be a long way from hazard and risk communication and to adequate safety behavior. Hazard and risk communication measures are deemed to fail, if cognitive dissonance, confirmation bias and the inadequacy of the deficit model are not taken into account.
- The presence of fragrances has a great economic impact on society. Estimated costs resulting from lost workdays and lost jobs due to illnesses from fragranced products in the workplace amounts to 14.8 billion Euro in Germany in 1 year. Costs, which the general public has to pay for the health care of affected persons would come on top of this.

Box 1 lists 10 recommendations on the way to reduce the number of self-reported adverse health effects caused by fragrances. The sequence proposed follows a hierarchical order. If non-chemical alternatives to fragranced products (1) are not feasible, fragrance-free spaces (2) and products (3) are recommended. If recommendations (2) and (3) are not feasible (4) and (5) are recommended and so on.

Box 1: Ten recommendations on how to reduce the number of self-reported adverse health effects caused by fragrances

1. Non-chemical alternatives to fragranced products.
2. Fragrance-free public spaces.
3. Fragrance-free products.
4. Substitution of hazardous ingredients by less hazardous substances.
5. Reduction of amounts used.
6. Identification and restriction of the substances leading to the adverse effects observed.
7. Labeling of all fragrance-containing mixtures and articles.
8. Mandatory lists of fragrance applied on all products and in scented indoor spaces, not only on personal care, washing and cleaning products.
9. Deletion of the exception clause in the CLP-regulation for cosmetic products.
10. Application of the hazard labels also for containers smaller than 125 mL.

The present COVID 19 pandemic, where people are using scented disinfectants and soap to a larger extent than before, leads to an increase of the exposure to fragrances especially in indoor air. It is to be expected that there will be more people who experience health effects due to fragrances under these conditions. This situation might stimulate a mind-shift in the public perception of fragrances, especially if severe cases receive broader attention or if celebrity role models support fragrance-free policies.

The issue of adverse health effects associated with fragrances has reached a dimension, which requires immediate improvements. It is time to act.

## Supplementary information

**Additional file 1.** SSI methodology.

**Additional file 2.** Survey methods.

**Additional file 3.** Calculation of economic costs.

## Data Availability

All data generated or analyzed during this study are included in this published article.

## Abbreviations

ESM: Electronic Supplementary Material
SSI: Survey Sampling International

## References

[1] Steinemann A, Klaschka U (2019). Exposures and effects from fragranced consumer products in Germany. Air Qual Atmos Health.

[2] Steinemann A (2019). International prevalence of fragrance sensitivity. Air Qual Atmos Health.

[3] Klaschka U (2020). This perfume makes me sick, but I like it. Representative survey on health effects associated with fragrances. Environ Sci Eur.

[4] Steinemann A (2019). Ten questions concerning fragrance-free policies and indoor environments. Build Environ.

[5] Attanasio R (2018). Communicating environmental sciences: public discourse and policy development. Integr Environ Assess Manage.

[6] Seiler T, Engwall M, Hollert H (2013). Lost in translation? Ways for environmental sciences to communicate about risk and research. Environ Sci Eur.

[7] Retzbach A, Marschall J, Rahnke M (2011). Public understanding of science and the perception of nanotechnology: the roles of interest in science, methodological knowledge, epistemological beliefs, and beliefs about science. J Nanopart Res.

[8] Rowe G, Wright G (2001). Differences in expert and lay judgments of risk: myth or reality?. Risk Anal.

[9] Steinemann A (2018). Exposures and effects from fragranced consumer products in Sweden. Air Qual Atmos Health.

[10] Steinemann A (2016). Fragranced consumer products: exposures and effects from emissions. Air Qual Atmos Health.

[11] Steinemann A (2017). Health and societal effects from exposure to fragranced consumer products. Prev Med Rep.

[12] Steinemann A (2018). Fragranced consumer products: sources of emissions, exposures, and health effects in the UK. Air Qual Atmos Health.

[13] Toncar M, Fetscherin M (2011). A study of visual puffery in fragrance advertising. Is the message sent stronger than the actual scent?. Eur J Marketing.

[14] Duftspender und Raumdüfte im Pflegeheim Duftmarketing Blog. http://www.duft-marketing.org/?p=275. Accessed 08 Mar 2020. **(in German)**

[15] Steinemann AC, MacGregor IC, Gordon SM (2011). Fragranced consumer products: chemicals emitted, ingredients unlisted. Environ Impact Assess Rev.

[16] Hartmann S, Klaschka U (2017). Interested consumers’ awareness of harmful chemicals in everyday products. Environ Sci Eur.

[17] Epp A, Hertel R, Böl G-F (2010). Chemie im Alltag: eine repräsentative Befragung deutscher Verbraucherinnen und Verbraucher.

[18] Lysdal SH, Johansen JD (2009). Fragrance contact allergic patients: strategies for use of cosmetic products and perceived impact on life situation. Contact Dermatitis.

[19] Haberland MF (2010). The power of scent: empirical field studies of olfactory cues on purchase behavior.

[20] Rimkute J, Moraes C, Ferreira C (2016). The effects of scent on consumer behaviour. Int J Consum Stud.

[21] DAAB. https://www.daab.de/. Accessed 08 Mar 2020

[22] https://www.voitair.de/referenzen/. Accessed 23 Jun 2020

[23] https://www.duftmarketing.de/de/referenzen.html. Accessed 23 Jun 2020

[24] https://www.umweltbundesamt.de/das-uba/was-wir-tun/foerdern-beraten/verbaendefoerderung/projektfoerderungen-projekttraeger/raumbeduftung-innenraumluftqualitaet. Accessed 26 Jun 2020 **(in German)**

[25] Statistisches Bundesamt (2020) Bruttoinlandsprodukt (BIP) in Deutschland von 1991 bis 2019. zit. nach de.statista.com. https://de.statista.com/statistik/daten/studie/1251/umfrage/entwicklung-des-bruttoinlandsprodukts-seit-dem-jahr-1991/#professional. Accessed 22 Jun 2020 **(in German)**

[26] Effertz T (2019). Die Kosten des Rauchens in Deutschland im Jahr 2018 – aktuelle Situation und langfristige Perspektive. Atemwegs- und Lungenkrankheiten. Atemwegs- und Lungenkrankheiten.

[27] Effertz T, Verheyen F, Linder R (2014). Die medizinischen Kosten schädlichen Alkohol- und Tabakkonsums in Deutschland – eine Analyse mittels GKV-Routinedaten. SUCHT.

[28] IKW (2019) Jahresbericht 2018/2019 Industrieverband Körperpflege- und Waschmittel. https://www.ikw.org/fileadmin/ikw/IKW/Pressebereich/IKW_Jahresbericht-2018_2019.pdf. Accessed 20 Mar 2020 **(in German)**

[29] Balda F (2019) Parfums und Duftprodukte - Werbeausgaben in Deutschland 2017. Statista. https://de.statista.com/statistik/daten/studie/196835/umfrage/werbeausgaben-fuer-parfums-und-duftprodukte-in-deutschland/. Accessed 08 Mar 2020 **(in German)**

[30] Pötschke-Langer M, Kahnert S, Schaller K et al. (2015) Tabakatlas. Publication by the DKFZ (German Cancer Research Center). https://www.dkfz.de/de/tabakkontrolle/download/Publikationen/sonstVeroeffentlichungen/Tabakatlas-2015-final-web-dp-small.pdf?m=1528122069&. Accessed 22 Jun 2020 **(in German)**

[31] Verbraucherfalle Parfüm - Vorsicht, Verbraucherfalle! - ARD Das Erste. ARD. https://www.daserste.de/information/ratgeber-service/vorsicht-verbraucherfalle/sendung/parfuem-folge-1-100.html. Accessed 08 Mar 2020 **(in German)**

[32] ECHA (2012) Communication on the safe use of chemicals: study on the communication of information to the general public. https://echa.europa.eu/documents/10162/13559/clp_study_en.pdf. Accessed 20 Mar 2020

[33] EC (2008). European Commission regulation No 1272/2008 of the European Parliament and of the Council of 16 December 2008 on classification, labelling and packaging of substances and mixtures. Off J Eur Union.

[34] Klaschka U, Rother H-A (2013). ‘Read this and be safe!’ Comparison of regulatory processes for communicating risks of personal care products to European and South African consumers. Environ Sci Eur.

[35] ECHA (2011) Special Eurobarometer 360: Consumer Understanding of Labels and the Safe Use of Chemicals. http://ec.europa.eu/public_opinion/index_en.htm. Accessed 20 Mar 2020

[36] Klaschka U (2010). Risk management by labelling 26 fragrances?: evaluation of Article 10 (1) of the seventh Amendment (Guideline 2003/15/EC) of the Cosmetic Directive. Int J Hyg Environ Health.

[37] Klaschka U (2012). Dangerous cosmetics—criteria for classification, labelling and packaging (EC 1272/2008) applied to personal care products. Environ Sci Eur.

[38] ECHA (2013) Flash Eurobarometer 361. Chemicals. http://ec.europa.eu/commfrontoffice/publicopinion/flash/fl_361_en.pdf. Accessed 02 Aug 2017

[39] Petersson McIntyre M (2013). Perfume Packaging. Seduction and Gender. CU.

[40] Rother H-A (2014). Communicating pesticide neurotoxicity research findings and risks to decision-makers and the public. NeuroToxicology.

[41] Lunny S, Nelson R, Steinemann A (2017). Something in the air but not on the label: a call for increased regulatory ingredient disclosure for fragranced consumer products. UNSWLJ.

[42] Klaschka U, Kolossa-Gehring M (2007). Fragrances in the environment: pleasant odours for nature?. Environ Sci Pollut Res Int.

