# Supplementary material for: Between attraction and avoidance: from perfume application to fragrance-free policies
Source: Environ Sci Eur. 2020 Jul 17;32(1):98. doi: 10.1186/s12302-020-00377-8 (PMC7366882; doi:10.1186/s12302-020-00377-8)
Supplement: Supplementary file 1 — Additional file 1. SSI methodology. [file 12302_2020_377_MOESM1_ESM.pdf]

Survey Sampling International (SSI)

Details on methodologies

1. Sample Blending (pages 2-11)
2. Answers to 28 Questions (pages 12-22)
3. Dynamix (pages 23-28)

For any questions for Survey Sampling International (SSI), please contact

Pete Cape, SSI USA, Knowledge Team E: Knowledge@SurveySampling.com
Juliana Tan, SSI AUS, Senior Account Executive E: Juliana.Tan@surveysampling.com

White Paper

Sample Blending: 1+1 > 2

This paper is based on a white paper which was first presented at the CASRO Panel Conference in February 2010, co-authored by Jackie Lorch and Kristin Cavallaro of SSI and Robert van Ossenbruggen, consultant.

Global DIY Mobile Online Phone Sample CATI

© Survey Sampling International 2014

SSI®

info@surveysampling.com | surveysampling.com

Multi-source samples have largely been considered a "necessary evil"—a requirement when no single source can handle a large or low incidence sampling need. Historically, researchers have shied away from using multiple sources—perhaps because sampling theory dictates the use of a defined, single population. However, in a changing world where online communication has moved away from e-mail, social media is playing an increasingly important role in opinion sharing and mobile phone is swiftly becoming the communication mode of choice, the traditional access panel paradigm is no longer sufficient.

In fact, the single most important characteristic for an unbiased sample is that it reflects the heterogeneity of the target population. Thus, increasing heterogeneity by blending samples from many different sources, each with their own unique bias should result in improving sample quality.
But only if we are able to identify this bias in order to control for it. If bias is correctly controlled for, the use of sample from multiple sources will improve the heterogeneity of the sampling pool and thus it's representativeness.

The only way to balance a multi-source sample is to get a real understanding of the source of its unique bias. One of the main problems with sampling from panels alone is that some people will never be in a panel. Just as a manufacturer of golfing equipment who conducts research solely among members of country clubs would be missing large segments of the target audiences, panels may be missing a distinct type of opinion-giver. While many people want to give their opinions, some shy away from the commitment of joining a panel. A blended approach incorporates panels, communities and groups with aligned interests. The whole Internet becomes the panel.

As well as being diverse, sample must be consistent and predictable. We must find a way to both increase diversity and deliver that diverse sample time after time. Multiple studies, including those by GfK, MKTG, Inc. and the ARF's Foundations of Quality initiative have shown that different sample sources have distinct characteristics, that there are many factors influencing these differences, that it is difficult to identify these factors but that controlling for demographics and other traditional balancing factors does not smooth out all of the variation. The ARF study identified recruitment techniques, (not just sources, but the recruitment approach), as well as the composition of the panel by longevity, reward methods, and other factors which haven't yet been fully researched and understood. In this paper we reveal the distinct characteristics of different sample sources and describe a way to smooth out between-source variation.

How Can a Sample Buyer Ensure a Consistent, Reliable Blend?

To ensure a consistent blend of sample, we can look at sources and see how they perform against external benchmarks, when available, but most research questions do not have readily available external benchmarks. Alternatively, we can use quotas by socio-demographics as we have always done to create samples which look alike and "look like" the general population. But demographics are often not the most helpful or relevant stabilizers.

Take the absurd example where an online survey is conducted using only respondents who visit a website devoted to fishing. Since fishing is a reasonably popular sport among all age groups, we could produce a sample from this site that looked "right" in terms of demographics. But what sort of

(continued)

© Survey Sampling International 2014

answers would they give? If the question is about brands of coffee bought, then their answers could well reflect the wider population—being interested in fishing doesn't affect the coffee you drink. But what if the questions were about media consumption? Anglers spend a lot of weekend time fishing, not watching TV. So the answers we get on TV viewing may not be anywhere close to the truth and you, the client, would never know this.

Using demographic quotas to ensure consistency works—as long as the stratification chosen is relevant to the topic of the questionnaire. And even when there is no correlation between, say, age and gender and perception of the product in question, the random nature of the sampling from a large population means that the chance of pulling a sample that has attitudes completely at odds with the last sample pulled is very small.

When using multiple online sources, however, you may be dealing with a number of relatively small universes that are not static and are possibly shrinking at each wave (especially if category resting is applied). Under these circumstances, it is much more likely that the true underlying constructs that go to define consumer choice in the category in question may differ in their prevalence in the sample pulled. It is not unusual for a product to be equally liked by both genders, across all age groups, regions and social classes—think Coke, for example.

Something, however, drives consumer preference for Coke over Pepsi. It is this "something," or a proxy for it, that needs to be controlled for or stratified on in the sampling process to ensure that two samples are truly comparable to one another, across time and across sources. Without it, the preference scores for Coke vs. Pepsi will fluctuate. In theory, the two samples balanced on the underlying "something" could be completely different in terms of age, gender, region and socio-economic factors and still come up with the "true" answer for Coke vs. Pepsi.

The two research studies reported in this paper are part of a research initiative Survey Sampling International (SSI) undertook to identify the components of this "something" and design a stabilization method to control the variation between sources which goes beyond socio-demographics. With an effective set of stabilization variables, identified at the individual participant level, it is not necessary to maintain exactly the same blend of sources from project to project. The number and proportion of sources can vary without causing variation in the results achieved.

If we can identify distinct psychographic, neurographic, and personality variables which are relevant to the way people answer surveys, we can control for them, and systematically broaden the diversity of our sample by finding sources which complement each other. For example it has been posited that people who enjoy participating in surveys from any source may have higher cognitive capabilities or a higher need for cognition (Cacioppo and Petty).

The first step in this process was to identify a broad set of factors which would define groups of people and potentially drive variance in behavior. We developed the questionnaire to include the major strands of thinking in relevant psychographic, neurographic, personality type, cognitive, learning and communication style. Elements included:

(continued)

© Survey Sampling International 2014

- Personality traits, using traditional rather than Jungian style
- Music preferences (Rentfrow)
- Cognitive ability (Kahneman and Frederick)
- Geographic/personality alignment (Gosling and Rentfrow)
- Social Values (There are a number of models; Schwarz values were chosen because they were freely available and had some available international benchmarks, having been used successfully in Europe.)
- Need for cognition measures (Cacioppo et al)
- Neurographics measures
- Propensity factors such as the propensity to participate, risk averseness, attitude to privacy, propensity to share and information usage
- Chronotypes, i.e. "lark" or "owl" personalities (Roenneberg)
- Disruption/orienting reflex measure and habituation disruption. (We considered these measures potentially insightful since they were not linguistically mediated. Examples used were background color changes and response time measurements, i.e. how much hesitation did a change in background color create?)

We chose each test as typical in class and in the absence of agreement from academics in the psychology of marketing as to the one definitive test in each discipline. We also chose them because, for some, external benchmarks exist, and they are, in general, less susceptible to cultural differences than other types of factors; any process we adopted needed to be international in scope and outlook. A number of additional measures were added. In all, 162 measures were tested.

Questions were created or adapted around each of the identified factors. These were tested against questions on topics such as technology adoption and usage, hobbies and interests, brand preference and loyalty, and reaction to advertisements—representing the most common types of general survey research that our clients are conducting today.

Sample came from the SSI US Survey Spot panel. As an aside, the questionnaires, at 34 minutes and 20 minutes, were longer than our ideal length. As we frequently preach about questionnaires being too long, we hesitated before placing them in field, especially as we offered no individual reward. However, the participant comments we received such as "Loved the variety of questions" and "The questions challenged me to look at myself," and the fact that the drop rate on the second study at 7.6% was considerably lower than our average drop rate, bolstered our belief that there is a difference between "actual" and "perceived" survey length. If a survey is compelling and a topic for which people have engagement (talking about themselves for example!), they have greater tolerance for a longer survey.

Two thousand completes were obtained in the study, which produced two major findings. First, many variables explained considerably more variance in the dependent variables than socio-demographics alone. Here are some examples:

(continued)

© Survey Sampling International 2014

		Explained variance with 16 best-performing independent variables	Explained variance with socio-demographics
When a new technology or product is invented that interests you, how soon after its release do you purchase?	7%	16%	2.3
How important to you are brand names when purchasing an item?	3%	8%	3.1
On how many of the past 5 weekdays have you read a newspaper?	10%	12%	1.2
Do you have a cell phone?	4%	7%	1.6
How frequently do you make or receive calls or texts on your cell phone?	13%	18%	1.4

Through iterative analysis techniques, each of the 162 variables we started with was tested on its power to "move the needle" on a variety of dependent variables. 16 variables were identified as a cluster which moved the needle most strongly.

Second, neither our cluster nor socio-demographics explained most of the variance. In other words, a person's presence in one or other of the clusters had little impact on whether they preferred the blue or the red shirt. Although it appeared that keeping this specific cluster balance constant would not provide gains which justified the additional complication of balancing on them every time we draw sample, this research study gave us intriguing hints about the potential of psychographic and neurographic measures to outperform socio-demographics as a sampling balancing mechanism.

The next phase of the research was, obviously, to re-test all this within a multi-sourced environment. In this phase, we benefitted greatly by working with Robert van Ossenbruggen, architect of the seminal 2006 NOPVO study. This study, which compared the performance of 19 panels in The Netherlands, was referenced in the design of the ARF Foundations of Quality study of 2009.

The sources used in the second study offered a diverse representation of the way people are brought into surveys and rewarded in the online environment:

- The SSI SurveySpot general population panel recruited online and rewarded with a variety of rewards, primarily sweepstakes and instant win prizes with some cash
- An SSI proprietary panel with a general population frame much of whom are recruited in person at shopping malls. Members have an affinity to their local mall and are rewarded with mall gift certificates.
- An SSI teen and young people panel, recruited to support gaming and entertainment research, and rewarded with prizes designed to appeal to a younger age group such as iTunes downloads

(continued)

© Survey Sampling International 2014

- A database of people who are rewarded for performing various activities online
- A general opt in access panel
- A "river" source
- Another general opt in access panel

Results from study two confirmed and expanded on results arrived at in study one. Three major findings were determined.

1. Different sources, as expected, produced different results. The differences were found across a wide range of topics and questions:

Sample sources*:	A	B	C	D	E	F	SD	Variance
Universalism as principle	5.75	5.49	6.02	5.90	5.80	5.52	1.72	0.044
Prayer in school, agree	2.92	3.03	2.50	2.84	3.07	2.93	1.44	0.042
Hedonism as principle	5.48	5.24	5.55	5.54	5.52	5.09	1.88	0.037
Tradition as principle	5.62	5.62	5.98	5.79	5.72	5.41	1.69	0.037
Trust internet transactions	2.93	2.62	3.11	2.86	2.84	3.03	1.29	0.029
See self as disorganized, careless	2.36	2.32	2.53	2.59	2.39	2.74	1.57	0.026
Traditional role of women	3.41	3.56	3.10	3.37	3.37	3.50	1.26	0.025
See self as critical, quarrelsome	3.11	3.12	2.83	3.11	3.07	3.32	1.64	0.025

* One source was removed for clarity on this chart because it has an inherently different demographic make-up than the others.

We saw differences across the seven sources in many of the areas commonly studied in market research including attitude to new technology, use of organic produce, use of coupons, shopping decision drivers (whether buy on price or quality, financial habits and daily newspaper readership).

2. Balancing on education, income, employment and marital status, (the samples were pre-balanced on age and gender) doesn't help us much in explaining the variance.

3. Using the psychological and neurographic variables does more to explain the variance, as we see in this summary chart for the entire study:

	No balancing	Balancing with socio-demographic variables	Balancing with psychographic, etc. variables
Between sample variation metric 1*	0.88	0.76	0.48
Improvement factor		1.15	1.83
Between sample variation metric 2**	18%	14%	9%
Improvement factor		1.29	1.92

*This metric was calculated as follows: 1. Means of IVs tabulated per source 2. Variance between sample means calculated 3. Sum of variances calculated.

**This metric was calculated by calculating % of subsample means deviating 0.1 SD from total score.

(continued)

© Survey Sampling International 2014

A Specific Example

"Willingness to try new things" was one of the variables with significantly different results across the seven sources. In looking at a simple cross tabulation of being willing to try new things and technology adoption, we see that people were less likely to adapt to new technology if they weren't open to trying new things (as we would expect). As we might also have predicted the willingness to try new things correlates with possession of technology such as a BlackBerry® cell phone or an MP3 player.\

To look at the effect of different levels of demographic variables and compare the effect of different levels of psychographic variables, we created a set of samples with different mixes—from a "mild mix" to an "extreme mix." When we mix the levels of different ages within samples, the differences in ownership of various new technologies does not change as can be seen in the following graph.

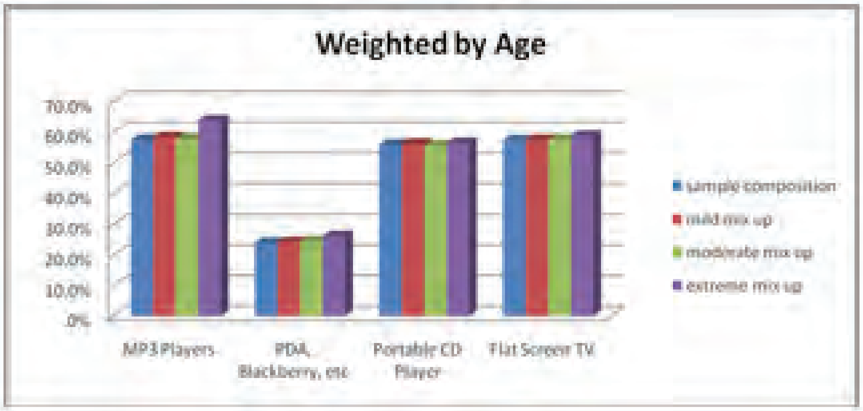

When we create a set of samples with different mixtures of levels of technology adoption, however, the resulting ownership of MP3 players, PDA, BlackBerry®, and Flat Screen TV varies significantly.

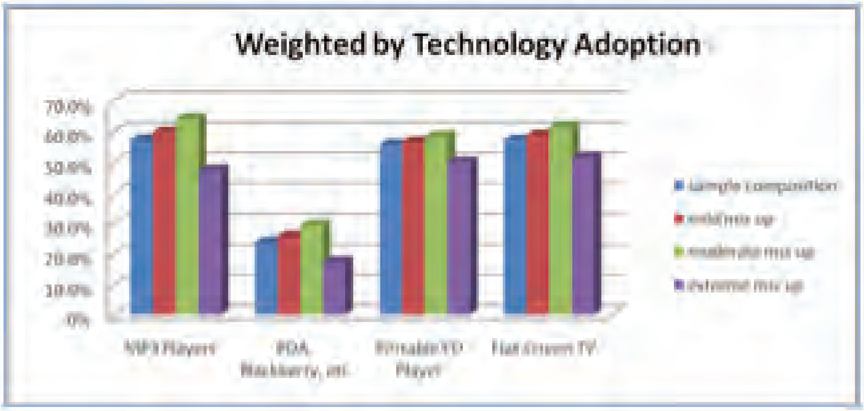

This is also the case when we create different samples with different mixtures of propensity to try new things.

(continued)

© Survey Sampling International 2014

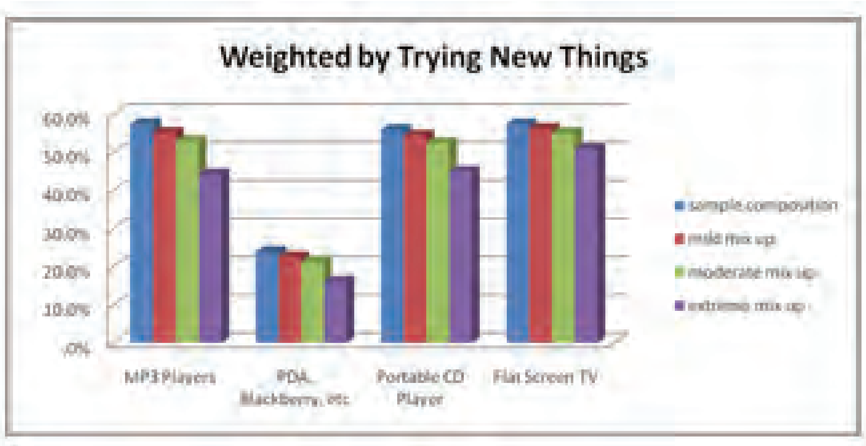

Again, this is important because the likelihood to try new things was one of the variables that was significantly different across many of the sources we tested.

Our advice from this example is to include a question about an individual's comfort level with technology when blending sample for research projects concerning technology.

This is one example of how we arrive at one element in a stabilization cluster. We expect to be using thousands of sources to create samples in the future, so we require a stabilization metric which will work at the individual person level, rather than the source level. We used data from this and other tests to refine the group of variables identified in the first test and tested the resultant calibration measure. We then created a set of questions (question clusters) which can be asked of any potential research participant before they enter a survey. SSI asks these questions of every nth respondent coming into SSI Dynamix. Since the questions focus on underlying characteristics, they need only be asked once, and, over time, the majority of SSI sample respondents have become profiled..

Viewing multiple sources of sample as a vast reservoir, the variable set is "state of the water" calibration measure. We use this measure when we dip our cup into the reservoir to test the water to monitor its health. The SSI Blend is monitored daily. Any significant new source is blend-profiled before being added to the reservoir to understand its impact on the overall blend, and, if necessary make adjustments to the inclusion plan for the new source.

The SSI Blend Put to the Test

After five years of testing more than 300 panels in 35 countries as part of their *Grand Mean Project*™, MKTG, Inc. have stated that only 11 panels demonstrated consistency through the Mktg Inc. *Consistent Track*™ audit.

SSI's online sample blend in the Netherlands, United Kingdom, France, Germany, Italy, Spain, Canada and the United States was awarded **highest honors for exceptional consistency in the audit.**

"No panel company **has achieved this level of excellence**," say the independent Sample Source Auditors™, a division of Mktg, Inc. "SSI has clearly demonstrated an ability to be **consistent in more markets than any other company."**

(continued)

© Survey Sampling International 2014

In Summary

1. Multi-sourcing is the future of online sampling; the world is changing and the traditional e-mail invitation access panel paradigm is no longer a fit.
2. Multi-sourced sample, incorporated into a single sample, source-certified, quality-controlled and managed for consistency provides superior reach and diversity compared to older panel models or individual sources.
3. We can keep multi-sourced sample consistent by pre-profiling research participants.
4. Pre-profiling must be approached in a new way; the way the industry has pre-profiled in the past with standard socio-demographics isn't adequate.
5. SSI has created a concise list of relevant variables proven over 5 years and a hundred thousand projects to deliver consistency and quality results.
6. Identification of bias and controlling for it in multi-sourced sample is a work in progress. The SSI Blend is continually monitored and adjusted to deliver consistency and support representivity.

Some Practical Steps for Blending Sample

1. Know your sources—understand how participants are recruited, where they come from, how they are rewarded, and means of communication.
2. Plan ahead and blend sample from the beginning of the project to avoid having to incorporate new sources midway through.
3. 3. Include some calibration questions, such as the example of a willingness to try new things, customized for both the type and topic of your research study.
4. 4. Discuss with your sample provider their techniques for blending sample. Ask if they use source smoothing as well as quality controls such as digital fingerprinting to ensure there is no duplication.

Finally, we are concerned with blending because we want to ensure that the opportunity to take surveys is placed in front of as large and diverse a population as possible. But the effort is in vain if our non-response and non-completion rates are so high that we introduce bias from those factors. We must also focus on making the questionnaire better for participants and ensuring the research experience is one that they will find, if not always thrilling, at least satisfying enough that they would be willing to do it again.

© Survey Sampling International 2014

References

Cacioppo, John T. and Richard E. Petty. “The Need for Cognition.” *Journal of Personality and Social Psychology*, 42, 116-131. 1982.

Cacioppo, J. T., Petty, R. E., Feinstein, J, A., & Jarvis, W. B. G. “Dispositional differences in cognitive motivation: The life and times of individuals varying in need for cognition.” *Psychological Bulletin*, 119, 197—253. 1996.

Rentfrow, P. J., & Gosling, S. D. “The do-re-mi’s of everyday life: The structure and personality correlates of music preferences.” *Journal of Personality and Social Psychology*, 84, 1236-1256. 2003.

Peter J. Rentfrow, Samuel D. Gosling, and Jeff Potter. “A Theory of the Emergence, Persistence, and Expression of Geographic Variation in Psychological Characteristics.” *Perspectives on Psychological Science* 3 (5) Pages 339 - 369. 2008.

Fallig, Michael and Derek Allen. “An Examination of strategies for Panel Blending” *Quirks* July 2009.

Foundations of Quality Knowledge Briefs 1, 2 and 3 *Advertising Research Foundation*. www.thearf.org. 2009.

Kahneman, Daniel and Shane Frederick, “Frames and Brains, Elicitation and Control of Response Tendencies.” *Trends in Cognitive Sciences*, Volume 11, number 2. Elsevier. 2006.

Schwarz, N., & G. L. Clore, “Feelings and phenomenal experiences.” *Social Psychology: Handbook of basic principles* (2nd ed., pp. 385-407). New York: Guilford. 2007.

Till Roenneberg, Till, Anna Wirz-Justice and Martha Merrow, “Life between Clocks: Daily Temporal Patterns of Human Chronotypes.” *Journal of Biological Rhythms*, Vol. 18, No. 1, 80-90. 2003.

More information about the NOPVO study can be found at http://www.nopvo.nl/english/english.htm

© Survey Sampling International 2014

ESOMAR 28

28 Questions to Help Research Buyers of Online Sample

The primary aim of these 28 Questions is to increase transparency and raise awareness of the key issues for researchers to consider when deciding whether an online sampling approach is fit for their purpose. Put another way, the aim is to help researchers to ensure that what they receive meets their expectations. The questions are also designed to introduce consistent terminology for providers to state how they maintain quality, to enable buyers to compare the services of different sample suppliers. Notes on the context of the questions explain why the questions should be asked and which issues researchers should expect to be covered in the answer.

These new questions replace ESOMAR's "26 Questions to Help Research Buyers of Online Samples.' ESOMAR has updated the text to recognize the ongoing development of techniques. While some of the questions remain constant, new questions have been added to incorporate new techniques and new technology in this area. In particular, this revision recognises the broad trend within the industry to build online samples from multiple sources rather than relying on a single panel.

It should be noted that these 28 Questions focus on the questions that need to be asked by those buying online samples. If the sample provider is also hosting the data collection, you will need to ask additional questions to ensure that your project is carried out in a way that satisfies your quality requirements.

The 28 Questions complement ESOMAR's Guideline to Online Research, which was revised in 2011 to add updated legal and ethical guidance and new sections on privacy notices, cookies, downloadable technology and interactive mobile.

COMPANY PROFILE

1. What experience does your company have in providing online samples for market research?

Context: This answer might help you to form an opinion about the relevant experience of the sample provider. How long has the sample provider been providing this service and do they have, for example, a market research, direct marketing or more technological background? Are the samples solely provided for third party research, or does the company also conduct proprietary work using their panels?

Survey Sampling International, LLC ("SSI"), founded in 1977, was the first company to make random sample available to researchers and invented the random sampling telephone methodologies which are still considered the gold standard today. SSI has 38 years of experince in the industry. With its roots in the methodology of random telephone sample, SSI is the only provider to offer sample across the full range of modes, including telephone (both fixed/landline and wireless/mobile), address-based, mail, face-to-face, online, mobile and mixed access and mixed mode sampling. SSI is therefore uniquely positioned to recommend the best methodology for each research project.

SSI offers the broadest reach in global sample available as well as global telephone data collection services. SSI partners with researchers to conduct more than 32 million surveys annually. The resulting deep sampling and data collection expertise enables SSI to provide consultation for each stage of the process—including sample methodology, questionnaire design, contact methods and rewards—ensuring that the right people complete the questionnaire carefully and attentively.

SSI has decades of experience with trackers, diaries, blogs, online bulletin boards and more, and is the sampling provider chosen by more than 2,500 satisfied customers including universities, political polling, consumer and business-to-business researchers. SSI provides sample across 100+ countries, has 30 offices spanning every time zone and staff fluent in 42 languages.

SAMPLE SOURCES AND RECRUITMENT

2. Please describe and explain the type(s) of online sample sources from which you get respondents. Are these databases? Actively managed research panels? Direct marketing lists? Social networks? Web intercept (also known as river) samples?

Context: The description of the types of sources a provider uses for delivering an online sample will provide insight into the quality of the sample

SSI's actively-managed proprietary panels across the globe are at the core of SSI's online sample. SSI improves the quality and representative nature of its proprietary panels by incorporating participants from partnership sources which SSI also actively manages. SSI's sample recruitment strategy is quite different from the simple "river" approach: participants are recruited via partnerships, invited via banners, invitations and messaging, then go through rigorous quality controls before being included in SSI panels.

3. If you provide more than one type of sample source: How are the different sample sources blended together to ensure validity? How can this be replicated over time to provide reliability? How do you deal with the possibility of duplication of respondents across sources?

Context: The variation in data coming from different sources has been well documented. Overlap between different panel providers can be significant in some cases and de-duplication removes this source of error and frustration for respondents.

Because sources are not only different from each other, but can also change over time, SSI uses a combination of personality and psychographic characteristics to understand and identify the underlying traits which make a difference in the way people answer survey questions. By asking participants a short set of key questions, SSI can control the characteristics of people within the sample. As a result, SSI is able to provide an exceptionally consistent sample blend measured by comparison with external benchmarks, including telephone sample studies and Industry Measures such as the Grand Mean. The Grand Mean auditors commented that "SSI has clearly demonstrated an ability to be consistent in more markets than any other company." The SSI Blend is continuously monitored and calibrated by a dedicated team of methodologists and analysts.

Each panelist in SSI's system is assigned a unique ID to track participation. SSI uses digital fingerprinting, to ensure that the same person does not take a survey more than once from the same device. In addition, SSI's suite of quality controls work to prevent fraudulent duplication from a single person who is using different devices. Among these controls, SSI employs Real Answer™ software from Imperium which identifies suspicious or duplicate answers to open-ended questions.

4. Are your sample source(s) used solely for market research? If not, what other purposes are they used for?

Context: Combining respondents from sources set up primarily for different purposes (like direct marketing for example) may cause undesirable survey effects.

SSI does not conduct any direct marketing or allow any of its proprietary panels to be used for direct marketing. SSI improves the breadth and representative nature of its sample by including additional partnership sources within its sample blend, including sources that engage in direct marketing activities. SSI's recruitment practice is to include multiple diverse sources to minimize bias. Any potential for source effect can be discussed at the sample planning stage.

5. How do you source groups that may be hard to reach on the internet?

Context: Ensuring the inclusion of hard-to-reach groups on the internet (like ethnic minority groups, young people, seniors, etc.) may increase population coverage and improve the quality of the sample provided.

SSI works with communities of interest to incorporate rare populations into the online sample blend. Recognizing that motivations may be different across different demographic groups, SSI's methodology allows participants to be rewarded in the way that makes most sense for them, so that the survey-taking experience will be satisfying and they will want to come back and take another survey in the future. By leveraging relationships with appealing, well-known brands, SSI uses loyalty mechanisms relevant to difficult-to-reach populations.

Questionnaire design has an increasingly important role in ensuring that harder-to-reach groups are included in the sample because more and more participants are choosing to take surveys on smartphones and tablets -- especially younger males and higher income individuals who can be more difficult to recruit to panels. To achieve the greatest reach across the entire online population it is essential to make all questionnaires mobile-friendly. These difficult-to-reach groups are excluded from the sample when the questionnaire is not mobile-friendly. SSI can advise on how to make questionnaires mobile-friendly and thus benefit from accessing the entire online sample frame represented by the SSI Blend.

SSI also realizes that online may not be the best solution for every project. SSI is methodology-neutral and can assist clients in understanding the tradeoffs of each method and recommend the best methodology.

6. If, on a particular project, you need to supplement your sample(s) with sample(s) from other providers, how do you select those partners? Is it your policy to notify a client in advance when using a third party provider?

Context: Many providers work with third parties. This means that the quality of the sample is also dependent on the quality of sample providers that the buyer did not select. Transparency is essential in this situation. Overlap between different providers can be significant in some cases and de-duplication removes this source of error and frustration for respondents. Providers who observe process standards like the ISO standards are required to give you this information.

All partners providing significant sample for SSI's sample blend must go through SSI's Certification Process before being used for any client project. SSI monitors vendor participant quality over time and does not engage - nor continue - with sources who cannot maintain consistently excellent scores.

The Certification Process includes the fielding of test surveys to assess the quality of the participants and their responses, including a number of proprietary quality control checks.

7. What steps do you take to achieve a representative sample of the target 7 population?

Context: The sampling processes (i.e. how individuals are selected or allocated from the sample sources) used are the main factor in sample provision. A systematic approach based on market research fundamentals may increase sample quality.

This process starts with exactly understanding the target population. Participants are selected from SSI's online sample blend, a consistently-managed, diverse and large frame. To minimize the risk of bias, SSI uses a three-stage randomization process in matching a participant with a survey they are likely to be able to complete. First, participants are randomly selected from SSI's panels to be invited to take a survey, and these participants are combined with others entering SSI's Dynamix™ sampling platform after responding to online messaging. A set of profiling questions is randomly selected for them to answer (these are methodologically correct questions, never affirmation questions) and upon completion, participants are matched with a survey they are likely to be able to take, using a further element of randomization. Questionnaires which are not designed to be mobile-friendly will be exposed to a limited sample frame only, since they will not be shown to the increasing number of participants who choose to take surveys on mobile devices.

8. Do you employ a survey router?

Context: A survey router is a software system that allocates willing respondents to surveys for which they are likely to qualify. Respondents will have been directed to the router for different reasons, perhaps after not qualifying for another survey in which they had been directly invited to participate, or maybe as a result of a general invitation from the router itself. There is no consensus at present about whether and how the use of a router affects the responses that individuals give to survey questions.

Yes, routing is a component of the SSI Dynamix sampling platform. SSI sees many benefits in the use of a well-designed and carefully-managed router. Routers can provide a much better participant experience, which results in a larger available sampling frame; they allow sample providers to better meet the low- incidence, scarce-population and short-field-time needs of today's researchers; and routers greatly minimize the risk of self-selections bias.

9. If you use a router: Please describe the allocation process with in your router. How do you decide which surveys might be considered for a respondent? On what priority basis are respondents allocated to surveys?

Context: Biases of varying severity may arise from the prioritization in choices of surveys to present to respondents and the method of allocation.

SSI's router was designed from the point of view of improving the participant experience, since the biggest source of dissatisfaction among participants is being turned away from surveys we have asked them to take. Since participant dissatisfaction leads to smaller sampling frames and risks participant fatigue and satisficing, a properly-designed router provides real quality benefits.

Multi-stage randomization is incorporated into the SSI Dynamix™ sample platform routing. Participants are randomly assigned to a series of profiling questions. Based on their answers, they are assigned, again using a randomization factor, to a survey they are likely to be able to take. Other factors considered in the assignment include the likelihood that they will be able to complete the survey and the characteristics of the specific study, including factors such as field time and incidence.

SSI's router is a parallel router design not a funnel router, meaning that if a person does not qualify for a study they are not directed down a funnel to the next study in line. Instead, they again return to the series of profiling questions. This ensures that the key step of randomization occurs before every survey.

10. If you use a router: What measures do you take to guard against or mitigate any bias arising from employing a router? How do you measure and report any bias?

Context: If Person A is allocated to Survey X on the basis of some characteristic then they may not be allowed to also do Survey Y. The sample for Survey Y is potentially biased by the absence of people like Person A.

While bias is inherent in every router, it is minimized when the router contains a large volume of diverse projects. A router with only a few projects, where the research topic or target population is closely correlated, has an increased risk of bias. The risk of bias is again minimized if the router is managed system-wide by a dedicated team and closely monitored for consistency.

SSI ran a test of 43 real client projects, both with the SSI DynamixTM sample platform router and without any router. No evidence was found that the router caused differences in the data obtained. In 36 of the 43 cases, there was no difference at all in the data, and in the remaining cases, the differences were attributable to other factors such as seasonality. A detailed White Paper on these tests is available from SSI.

Several SSI methodologists and analysts have been invited to take part in developing Industry Best Practices for routers and are active on Industry Task Forces and Committees on this topic.

The potential for bias introduced by routers must be weighed against biases occurring in a non-router environment, within which participants self-select from a number of individual survey invitations in their inbox. With a router, that self-selection bias, and additional potential biases caused by invitation wording, reward or survey topic are absent.

11. If you use a router: Who in your company sets the parameters of the router? Is it a dedicated team or individual project managers?

Context: It may be necessary to try to replicate your project in the future with as many of the parameters as possible set to the same values. How dificult or easy will this be?

This responsibility and ownership is firmly in the hands of a dedicated team; individual project managers do not control the router. This is important because integrity of the sample must override any immediate demands of an individual project.

Since SSI has a sample blend which is controlled for consistency by underlying characteristics of participants, and the consistency of the blend is constantly monitored and calibrated using external benchmarks, clients can expect consistent sample over time. In addition to blend controls, the combination of source types is also carefully monitored for tracking studies.

12. What profiling data is held on respondents? How is it done? How does this differ across sample sources? How is it kept up-to-date? If no relevant profiling data is held, how are low incidence projects dealt with?

Context: The usefulness to your project of pre-profiled information will depend on the precise question asked and may also depend on when it was asked. If real time profiling is used, what control do you have over what question is actually asked?

SSI holds this information on all participants. The methodology and process is the same, whatever the sample source. Profiling is important in providing a good participant experience by avoiding repetitive questions. SSI selects include

ailments, hobbies and lifestyles, product ownership, media consumption, auto ownership, travel, shopping habits, purchase intent by category, business titles and responsibility, employer profile information as well as detailed demographic and geographic information.

SSI fields only methodologically sound profiling questions, not affirmation questions, and the precise wording of any profiling question is always available upon request. In the tradeoff between profiling accuracy and excluding eligible participants, SSI will always err on the side of ensuring all eligible participants are available for selection.

Each question is stored in a library, managed by a dedicated team, and each question has an expiration date. For example, a question asking if someone likes to play golf will not be updated as frequently as one asking if someone has a sports injury. Once again, there is a tradeoff between burdening participants with too many profiling questions and maintaining updated data. The collection date range for any profile item is configurable and can be discussed at the sample planning stage.

13. Please describe your survey invitation process. What is the proposition that people are offered to take part in individual surveys? What information about the project itself is given in the process? Apart from direct invitations to specific surveys (or to a router), what other means of invitation to surveys are respondents exposed to? You should note that not all invitations to participate take the form of emails.

Context: The type of proposition (and associated rewards) could influence the type of people who agree to take part in specific projects and can therefore influence sample quality. The level of detail given about the project may also influence response.

SSI uses invitations of all types to bring in people with a diversity of motivations to take part in research. These include e-mail invitations, telephone alerts, banners and messaging on SSI panel community sites. The messages themselves are also varied, including invitations to give your opinion, win a prize, earn cash or prizes or let your voice be heard. A diversity of motivation contributes to high-quality sample.

To avoid self-selection bias, specific project details are not generally included in the invitation. Rather, participants are invited to "take a survey." The details are disclosed later, when a survey has been selected within the system.

14. Taking part in your surveys. How does this differ by sample source, please describe the incentives that participants are offered for by interview length, by respondent characteristics?

Context: The reward or incentive system may have an impact on the reasons why people participate in a specific project and these effects can cause sample bias.

SSI offers great diversity in incentives as another means to increase diversity of sample frames. Some people are motivated by cash or points, prizes or sweepstakes, or by being able to donate to charity. Others are motivated by the chance to make a difference, make their voice heard, have fun taking a survey, helping out, or having a say in the products and services of the future. Others are motivated by learning opportunities provided by the survey, or by the promise of receiving information. SSI aims to respond to all of these individual motivations, in order to provide a sample which is diverse and as representative as possible of the target population.

Rewards offered may vary by survey length and the characteristics of the population being targeted. SSI uses a reasonable level of reward based on the amount of effort required, the population, and appropriate regional customs.

SSI continues to invest in research-on-research into the motivations of online research participants, and continually adjusts its reward offerings based on these findings, and on current academic thinking about motivation and industry best practices.

15. What information about a project do you need in order to give an accurate estimate of feasibility using your own resources?

Context: The "size" of any panel or source may not necessarily be an accurate indicator that your specific project can be completed or completed within your desired time frame.

We will need to know 1) Who you wish to speak to; 2) What you will ask of them and 3) How much time is available to gather the data. The first item incorporates geography, demographics, incidence and quota structures and the number of completed interviews required; the second covers the length of the survey, any special tasks required, the questionnaire design and completion difficulty for the participants; the third defines the fielding period, which will impact feasibility.

16. Do you measure respondent satisfaction? Is this information made available to clients?

Context: Respondent satisfaction may be an indicator of willingness to take future surveys. Respondent reactions to your survey from self-reported feedback or from an analysis of suspend points might be very valuable to help understand survey results.

Yes, this is measured for every participant for every completed interview. The results are made available at the end of each project as a standard part of SSI's "Project Debrief Pack." SSI supports high quality

questionnaire design by underwriting a series of awards called the QUEST Awards. These awards honor companies who create high quality questionnaires. The awards are presented every year at the ESOMAR Annual Congress..

SSI's Engagement Team is dedicated to providing a positive participant experience. The team responds quickly to participant inquiries and takes immediate action to resolve any issues of dissatisfaction.

17. What information do you provide to debrief your client after the project has finished?

Context: One should expect a full sample provider debrief report, including gross sample, start rate, participation rate, drop-out rate, the invitation/contact text, a description of the field work process, and so on. Sample providers should be able to list the standard reports and metrics that they make available.

SSI's Standard Project Debrief Pack provides basic project information. Additional information is stored for approximately two years after the completion of the project and is available on request.

DATA QUALITY AND VALIDATION

18. Who is responsible for data quality checks? If it is you, do you have procedures in place to reduce or eliminate undesired survey behaviours, such as (a) random responding, (b) illogical or inconsistent responding, (c) overuse of item non-response (e.g. "Don't Know") or (d) speeding (too rapid survey completion)? Please describe these procedures.

Context: The use of such procedures may increase the reliability and validity of the survey data.

Sources used by SSI undergo a Certification Program which includes a series of quality control questions fielded with the source's sample. Sources whose participants do not perform well are not included in SSI's sample. Quality control questions are incorporated into the questions which participants see as they are being profiled. SSI also works closely with clients, marking the ID of any participant who has been reported to SSI as a potential problem participant.

Both SSI's own research-on-research and multiple industry studies have concluded that questionnaire design is the biggest factor in poor quality response. For example, the ARF Foundations of Quality Study tested 17 online panel sources and found that bad participant behavior is 6 times as likely to happen in a long survey compared to a short one. SSI can provide consultation on the questionnaire designs most likely to provide a good participant experience and maximize attention.

If quality control questions are used incorrectly, they risk introducing bias to the data. SSI can provide advice on the best type of questions to use and how to use them, based on our own research and industry best practices.

19. How often can the same individual be contacted to take part in a survey within a specified period whether they respond to the contact or not? How does this vary across your sample sources?

Context: Over solicitation may have an impact on respondent engagement or on self-selection and non-response bias.

Solicitation limits vary across the hundreds of sources which make up SSI's sample stream. Some restrictions are "hard" limits, others "soft" guidelines. This diversity of solicitation level increases the diversity of sample and improves its ability to reflect the target population. Restricting solicitation, however, must be weighed against the risk of bias in excluding certain people from a survey solely based on their receipt of a previous solicitation. The sample blend is closely monitored and tested for consistency to ensure that neither solicitation frequency nor any other factor is causing unexpected change.

20. How often can the same individual take part in a survey within a specified period? How does this vary across your sample sources? How do you manage this within categories and/or time periods?

Context: Frequency of survey participation may increase the risk of undesirable conditioning effects or other potential biases.

Participation limits vary across SSI's panels. Some restrictions are "hard" limits, others "soft" guidelines. This diversity of participation level increases the diversity of sample and improves its ability to reflect the target population. Restricting participation, however, must be weighed against the risk of bias in excluding certain people from a survey solely

based on their previous participation. Panels are closely monitored and tested for consistency to ensure that neither resting nor any other factor is causing unexpected change.

Participants can be excluded from projects based on previous participation in or completion of any specific previous study on request.

21. Do you maintain individual level data such as recent participation history, date of entry, source, etc., on your survey respondents? Are you able to supply your client with a project analysis of such individual level data?

Context: This type of data per respondent, including how the total population is defined and how the sample was selected and drawn, may increase the possibilities for analysis of data quality.

Yes, panelist data is maintained and available on request until an account is terminated.

22. Do you have a confirmation of respondent identity procedure? Do you have procedures to detect fraudulent respondents? Please describe these procedures as they are implemented at sample source registration and/or at the point of entry to a survey or router. If you offer B2B samples, what are the procedures, if any?

Context: Confirmation of identity can increase quality by decreasing multiple entries, fraudulent panellists, etc.

SSI uses several fraud detection/fraud prevention techniques and tools to assist in ensuring the quality of survey data. These include:

- Engaging in data validation techniques both internally and through third parties
- Implementation of Imperium Relevant IDTM safeguards, including Real AnswerTM software providing open end quality control checks
- Device fingerprinting, which enables SSI and/or a third party service provider to identify a computer or device that participates in a survey more than once
- Two-factor authentication at time of reward redemption, similar to controls used by banks, which removes the incentive for most fraudulent behavior, since no reward can be retrieved without an offline confirmation.

Following the success of the techniques SSI has developed, SSI has been consulted by other sample providers and panel owners for guidance in combating this industry-wide issue.

POLICIES AND COMPLIANCE

23. Please describe the 'opt-in for market research' processes for all your online sample sources.

Context: The opt-in process indicates the respondents' relationship with the sample source provider. The market generally makes a distinction between single and double opt-in. Double opt-in refers to the process by which a check is made to confirm that the person joining a panel or database wishes to be a member and understands what to expect (in advance of participating in an actual survey for a paying client).

SSI panellists have agreed to join an SSI panel(s), through an opt-in registration process. Prospective panellists complete

a registration process to join an SSI panel and agree to SSI's privacy policy and terms and conditions. By becoming a panel member, they are agreeing to participate in survey research. Individuals engaged by or on behalf of SSI from or through third party sources demonstrate their consent to participate in survey research by entering the SSI Dynamix™ system and completing a survey.

24. Please provide a link to your Privacy Policy. How is your Privacy Policy provided to your respondents?

Context: Not complying with local and international privacy laws might mean the sample provider is operating illegally. An example privacy policy is given in the ESOMAR Guideline for Online Research.

Click to view SSI's Privacy Policy. SSI's General Counsel periodically reviews the privacy policy. A link to the policy is located on each page where personal information is collected. In addition, new panelists are required to consent to the policy during the panel enrollment process. SSI staff serve on industry committees working to maintain and develop best practices for privacy. SSI strives to conform its privacy practices to applicable laws, codes, and regulations, and the codes and standards of market and opinion survey research associations, including CASRO and ESOMAR.

25. Please describe the measures you take to ensure data protection and data security.

Context: The sample provider usually stores sensitive and confidential information on panellists and clients in databases. These data need to be properly secured and backed-up, as does any confidential information provided by the client. The sample provider should be able to provide you with the latest date at which their security has been evaluated by a credible third-party.

All panellist and participant information is secured via industry standard firewalls and stringent IT security policies and procedures. All computer equipment (servers, SANs, switches, routers, etc.) are redundant and are located in secure, environmentally controlled data centers with 24/7 monitoring. Access is restricted and requires authorization. Access to participant data is restricted by password and staff job function and is limited to secure company networks or secure VPN. Databases and associated backup files access is restricted by IT job function and role. Password-protected database roles further restrict data access and force any data modification to be done through the application layer only. All database connections are logged. Web traffic does not directly access the database and database requests are reversed proxy via an application server to the database.

SSI staff adhere to strict guidelines to prevent sharing of any information across projects or clients. For example, if a project moves from one research provider to another, no information will be shared to the second provider without the express written permission of the original research sponsor. All SSI employees must sign a confidentiality agreement upon joining the company which outlines the employee's obligations to protect company and client confidential information.

26. What practices do you follow to decide whether online research should be used to present commercially sensitive client data or materials to survey respondents?

Context: There are no foolproof methods for protecting audio, video, still images or concept descriptions in online surveys. In today's social media world, clients should be aware that the combination of technology solutions and respondent confidentiality agreements are "speed bumps" that mitigate but cannot guarantee that a client's stimuli will not be shared or described in social media.

SSI is highly experienced in supporting research projects with extremely sensitive material and regularly provides consultation to researchers on best practices in this area. There are a number of techniques which can discourage leaks, including disabling of copying and screen grabs, removal of images after a timed period, and special wording to the participant along with an "I agree not to share information." However, there is no guarantee that sensitive information can

be kept confidential online (it is easy to take a photo of the screen with a cell phone for example), and SSI may recommend an in-person interview as a better option for extremely sensitive material.

27. Are you certified to any specific quality system? If so which one(s)?

Context: Being certified may require the supplier to perform tasks in a pre-determined manner and document procedures that should be followed.

SSI is ISO Certified to Standard 20252, the standard for Market, Opinion and Social Research in its Sydney, Australia office.

SSI's telephone methodology, which was the foundation for its online sampling methodology, has been audited and verified by the Media Ratings Council. SSI is the recipient of multiple product and service awards such as the Gallup Premier Partner Award (SSI has won several times). SSI is an official Preferred Partner for leading research organizations across the globe. Following the success of fraud detection/ fraud prevention the techniques SSI has developed, SSI has been consulted by other sample providers and panel owners for guidance in combating this industry-wide issue.

28. Do you conduct online surveys with children and young people? If so, do you adhere to the standards that ESOMAR provides? What other rules or standards, for example COPPA in the United States, do you comply with?

Context: The ICC/ESOMAR International Code requires special permissions for interviewing children. These are described in ESOMAR's Guideline for Online Research. In the USA, researchers must adhere to the requirements of the Children's Online Privacy Protection Act (COPPA). Further information on legislation and codes of practice can be found in Section 6 of ESOMAR's Guideline for Online Research.

SSI advises clients to invite children and young people through parents or legal guardians and seek parental consent. In addition, we do not collect personal data from children under 13 in the United States so no COPPA compliance is required for our panel participants.

White Paper

Understanding Data Quality in Survey Results with Dynamix™

By Pete Cape, *Global Knowledge Director and* ***Kristin Cavallaro,*** *Knowledge Specialist*

© Survey Sampling International, 2014

White Paper: Understanding Data Quality in Survey Results with Dynamix™

Authors: ***Pete Cape, Global Knowledge Director & Kristin Cavallaro, Knowledge Specialist***

TABLE OF CONTENTS

Why Dynamix™ was developed 4

Executive summary 6

How Dynamix™ works 7

Objectives, measurements and considerations 8

Test framework 12

Detailed results: Non–Coverage errors 14

Detailed results: Over/under reporting 38

Detailed results: Halo and question order effects 41

Appendix 1: Full results, tests where no differences found 46

WHY DYNAMIX™ WAS DEVELOPED

The world is changing and this is true in the area of communication and information sharing more than almost any other. In a world where e-mail is no longer the currency of communication online and use of social media, gaming, and constant communication-on-the-go is a way of life for many, traditional access panel models are no longer equipped to sustain high quality research results into the next decade.

SSI's Dynamix™ was created to meet two primary needs: the need of research participants to be communicated with on their terms and presented with an engaging, pleasant experience; and the need for diverse, robust yet consistent samples to support high quality research data.

Dynamix™ represents a paradigm shift in sampling. It is an integrated platform with four major components:

1. **Reach** – combining the control of an access panel with the vast reach of the internet
2. **Experience** – a method for contacting and engaging research participants which respects their time, avoiding repetitive screenout experiences and so bringing them into every survey fresher and more prepared to be attentive, careful opinion-sharers
3. **Integrity** – sampling riguor is built into each stage of the system
4. **Engagement** – Dynamix™ provides a research participant with a survey they can take at the time they want to take it.

The SSI Dynamix™ methodology has many advantages from the respondent's point of view. People are no longer sampled for individual projects but offered one of many they may qualify for at the time at which they wish to do a survey.

Screen-outs are reduced, reducing the temptation to cheat into surveys and reducing dissatisfaction with the panel process. The number of emails in the panelist inbox will be reduced, possibly to a number matching their preference for invitation volume.

It also has advantages from the methodological point of view. It removes the self-selection bias inherent in traditional contact methods because participants are presented with only one survey at a time and can't "opt in" based on the attractiveness of the reward or topic. They also have no visibility into other survey opportunities available so have less incentive to "switch" mid-survey, leading to lower drop rates.

Profiling to determine qualification is done in real time so participants' profile data is fresher.

Multiple levels of randomisation have been built into the system. A participant who enters the system may potentially qualify for all the different projects currently live based on their known demographic or other characteristics. Ten refinement questions, representing ten of these potential projects, are randomly selected and presented to the participant. Based on their answers to these questions, may qualify for

anywhere from 0 to 10 of the 10 survey further level of randomisation is embedded in the system which selects the best survey for them to take at that moment.

After they have completed, or screened out of, the survey Dynamix™ sent them to, they are thanked and rewarded. The system then reviews again, in real time, how many projects they could potentially qualify to take. A new set of questions is randomly selected for them, and again, a random component is used to assign them to the best survey for them to take at that moment.

It is important to note that while Dynamix™ brings many changes, many underlying processes and procedures have stayed the same and work in the same way under the Dynamix™ system. These include the ability to deduplicate at multiple levels, participation controls, the SSI Verify system controlling overlap, duplication and providing quality checks, as well as other quality control and verification procedures.

The paradigm shift represented by Dynamix™ does raise a number of methodological issues that need to be examined and quantified to ensure Dynamix™ itself does not introduce any bias into sample that will materially affect the data.

The purpose of this document is to share results and analysis from the most extensive test to date of real projects run on the Dynamix™ platform. This test was part of the year-long research and development project to create Dynamix™, which in total encompassed 12,000 people hours and a seven-figure investment. Further tests are being planned and full details will again be shared once those are completed.

EXECUTIVE SUMMARY

The changes in process brought about by Dynamix™ beg a number of questions that form the primary objectives of the test.

1. How does the process of asking "refinement" questions (the "Refinement Questionnaire") before participants enter a survey chosen for them affect or cause non-coverage errors, over- or under- qualification errors or errors caused by halo or question order effects?

2. Does sending a general e-mail invitation to cover multiple surveys compared to the one-e-mail-per-survey approach change qualification rates?

The test is the first of many and was conducted in the US only with the SurveySpot panel. Further tests will be run with other SSI panels in all regions, but there is no known reason why these results would not be applicable to other countries. The test simulated the Dynamix™ system by re-running a diverse group of real projects originally run on SSI's system pre-Dynamix™.

Of the 39 surveys analyzed, 27 showed no statistically significant differences at all at the 95% confidence level. (Please see Appendix 1.)

For the remaining 12 we were able to find the key driver of the difference, which was not attributable to bias introduced by any aspect of the Dynamix™ system. In the remaining two cases we were unable to confirm the reason for the difference, but could put forward reasonable hypotheses which, again, were not attributable to the Dynamix™ system.

In summary, whether or not we were able to find the reason for the difference, in all cases it was not Dynamix™ that had caused it.

Our conclusion is that SSI client projects can safely be moved to the Dynamix™ platform without concern that the data will shift as a result of any component of the system. We recommend that all projects immediately start using the system in order to benefit from the improved methodology and respondent experience which Dynamix™ offers.

HOW DYNAMIX™ WORKS

The diagram below illustrates how a respondent may arrive at "survey a" under the previous paradigm and under
Dynamix™.

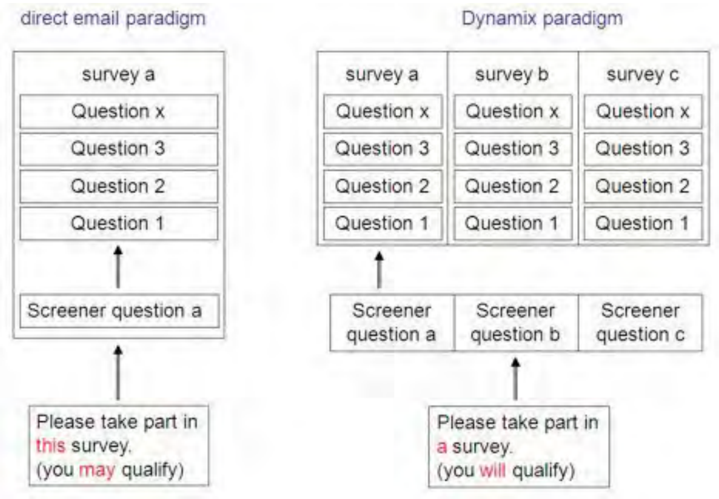

In Dynamix™ all the screener questions are gathered together in one survey which we call the Refinement Survey. An example of a refinement survey is below:

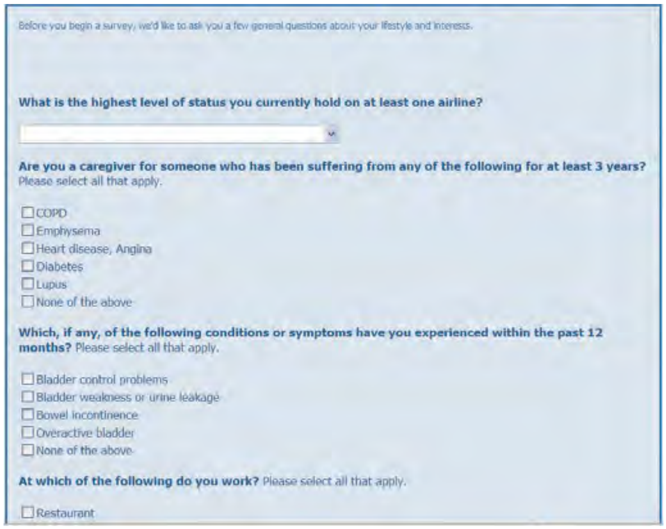

Before you begin a survey, we'd like to ask you a few general questions about your lifestyle and interests.

What is the highest level of status you currently hold on at least one airline?

Are you a caregiver for someone who has been suffering from any of the following for at least 3 years? Please select all that apply.

- ☐ COPD
- ☐ Emphysema
- ☐ Heart disease, Angina
- ☐ Diabetes
- ☐ Lupus
- ☐ None of the above

Which, if any, of the following conditions or symptoms have you experienced within the past 12 months? Please select all that apply.

- ☐ Bladder control problems
- ☐ Bladder weakness or urine leakage
- ☐ Bowel incontinence
- ☐ Overactive bladder
- ☐ None of the above

At which of the following do you work? Please select all that apply.

- ☐ Restaurant

Only after these questions have been completed does the system allocate a survey to the person.

Within Dynamix™ it is possible for a respondent to start and/or more than one survey in a single session. Maximum session lengths are set to minimize respondent fatigue and to help prevent the satisificing behaviour associated with long interview lengths.

For the full white paper and additional details, please contact SSI (information on the first page of this file).
