# Supplementary material for: Between attraction and avoidance: from perfume application to fragrance-free policies
Source: Environ Sci Eur. 2020 Jul 17;32(1):98. doi: 10.1186/s12302-020-00377-8 (PMC7366882; doi:10.1186/s12302-020-00377-8)
Supplement: Supplementary file 3 — Additional file 3. Calculation of economic costs. [file 12302_2020_377_MOESM3_ESM.docx]

**Calculation of economic costs due to fragrance exposure in the workplace on the basis of the survey data**

(Survey data are highlighted in green)

(Results are highlighted in red)

Overview and final results:

| General Population | 61 of 1102  5.5% |
| --- | --- |
| Fragrance Sensitive | 49 of 219  22.4% |
| Population Affected (81.45 Million inhabitants in Germany) | 4,48 Million (5.5% von 81,45 Million) |
| Lost workdays | 33.05 Million |
| Lost workdays per person | 7,4 (33.05 Million /4.48 Million=7,4 ) |
| Personal economic costs | 14,8 * 10^9^ Euro |

Calculation of lost workdays:

| **People affected** | **61** |
| --- | --- |
| **Lost 1 to 2 workdays** | **24** |
| Lower bound = 1 | 24 (24*1) |
| Mid-range = 1,5 | 36 (36*1,5) |
| Upper bound = 2 | 48 (48*1,5) |
|  |  |
| **3 to 6** | **18** |
| Lower bound = 3 | 54 |
| Mid-range = 4,5 | 81 |
| Upper bound = 6 | 108 |
|  |  |
| **7 to 14** | **11** |
| Lower bound = 7 | 77 |
| Mid-range = 10,5 | 115,5 |
| Upper bound = 14 | 154 |
|  |  |
| **15 to 30** | **3** |
| Lower bound = 15 | 45 |
| Mid-range = 22,5 | 67,5 |
| Upper bound = 30 | 90 |
|  |  |
| **Over 30** | **5** |
| Lower bound =30 | 150 |
| Mid-range = 30 | 150 |
| Upper bound = 30 | 150 |

|  |  |  | Days |
| --- | --- | --- | --- |
| **Sum over Lower bounds** | 350 | Sum lower / People affected * Population affected Germany  350/61*4.48*10^6= 2.57*10^7 | 25,7 Million |
| **Sum over mid-ranges** | 450 | Sum mid/ People affected * Population affected Germany  450/61*4.48*10^6= 3.31*10^7 | 33,05 Million |
| **Sum over upper bounds** | 550 | Sum upper / People affected * Population affected Germany  550/61*4.48*10^6= 4.04*10^7 | 40,4 Million |

Calculation of personal economic costs:

| **People affected** | **61** |
| --- | --- |
| **Less than 1000€** | **27,9 %** |
| Lower bound = 1 | 1,25E+06 (4,48 Million/100*27,9*1) |
| Mid-range = 500 | 6,25E+08 (4,48 Million/100*27,9*500) |
| Upper bound = 1000 | 1,25E+09 |
|  |  |
| **1000 to 10000€** | **31,1 %** |
| Lower bound = 1000 | 1,25E+09 |
| Mid-range = 5500 | 6,87E+09 |
| Upper bound = 10000 | 1,25E+10 |
|  |  |
| **More than 10000€** | **16,4%** |
| Lower bound = 10000 | 7,35E+09 |
| Mid-range = 10000 | 7,35E+09 |
| Upper bound = 10000 | 7,35E+09 |

| **Sum over Lower bounds** | 8,60E+09 |  |
| --- | --- | --- |
| **Sum over mid-ranges** | 1,48E+10 | = 6,25E+08+ 6,87E+09 + 7,35E+09 |
| **Sum over upper bounds** | 2,11E+10 |  |

The calculation was effectuated following the same procedure as in

Steinemann A (2019) International prevalence of fragrance sensitivity. Air Qual Atmos Health 12:891–897. https://doi.org/10.1007/s11869-019-00699-4
